# Comparative Effectiveness of Digital Cognitive Behavioral Therapy vs Medication Therapy Among Patients With Insomnia

**DOI:** 10.1001/jamanetworkopen.2023.7597

**Published:** 2023-04-11

**Authors:** Menglin Lu, Yaoyun Zhang, Junhang Zhang, Songfang Huang, Fei Huang, Tingna Wang, Fei Wu, Hongjing Mao, Zhengxing Huang

**Affiliations:** 1 Zhejiang University, Hangzhou, China; 2 Alibaba Damo Academy, Hangzhou, China; 3 Hangzhou Seventh People’s Hospital, Hangzhou, China; 4 Hangzhou slan-health Co, Ltd, Hangzhou, China

## Abstract

**Importance:**

Although digital cognitive behavioral therapy for insomnia (dCBT-I) has been studied in many randomized clinical trials and is recommended as a first-line treatment option, few studies have systematically examined its effectiveness, engagement, durability, and adaptability in clinical settings.

**Objective:**

To evaluate the clinical effectiveness, engagement, durability, and adaptability of dCBT-I.

**Design, Setting, and Participants:**

This retrospective cohort study was conducted using longitudinal data collected via a mobile app named Good Sleep 365 between November 14, 2018, and February 28, 2022. Three therapeutic modes (ie, dCBT-I, medication, and their combination) were compared at month 1, month 3, and month 6 (primary). Inverse probability of treatment weighting (IPTW) using propensity scores was applied to enable homogeneous comparisons between the 3 groups.

**Exposures:**

Treatment with dCBT-I, medication therapy, or combination therapy according to prescriptions.

**Main Outcomes and Measures:**

The Pittsburgh Sleep Quality Index (PSQI) score and its essential subitems were used as the primary outcomes. Effectiveness on comorbid somnolence, anxiety, depression, and somatic symptoms were used as secondary outcomes. Cohen *d* effect size, *P* value, and standardized mean difference (SMD) were used to measure differences in treatment outcomes. Changes in outcomes and response rates (≥3 points change in PSQI score) were also reported.

**Results:**

A total of 4052 patients (mean [SD] age, 44.29 [12.01] years; 3028 [74.7%] female participants) were selected for dCBT-I (n = 418), medication (n = 862), and their combination (n = 2772). Compared with the change in PSQI score at 6 months for participants receiving medication alone (from a mean [SD] of 12.85 [3.49] to 8.92 [4.03]), both dCBT-I (from a mean [SD] of 13.51 [3.03] to 7.15 [3.25]; Cohen *d*, −0.50; 95% CI, −0.62 to −0.38; *P* < .001; SMD = 0.484) and combination therapy (from a mean [SD] of 12.92 [3.49] to 6.98 [3.43]; Cohen *d*, 0.50; 95% CI, 0.42 to 0.58; *P* < .001; SMD = 0.518) were associated with significant reductions; dCBT-I had a comparable effect as combination therapy (Cohen *d*, 0.05; 95% CI, −0.05 to 0.15; *P* = .66; SMD = 0.05), but showed unstable durability. Outcomes of dCBT-I improved steadily and rapidly during the first 3 months, and then fluctuated. The response rates with dCBT-I and combination therapy were higher than with medication. Changes in secondary outcomes indicated statistically significant benefits from dCBT-I and combination therapy. The results of subgroup analysis were consistent with the main findings, demonstrating the superiority of dCBT-I vs medication therapy in various subpopulations.

**Conclusions and Relevance:**

In this study, clinical evidence suggested that combination therapy was optimal, and dCBT-I was more effective than medication therapy, with long-term benefits for insomnia. Future studies are needed to analyze its clinical effectiveness and reliability in distinct subpopulations.

## Key Points

**Question:**

Can clinical data be used to assess the effectiveness, durability, engagement, and adaptability of digital cognitive behavioral therapy for insomnia (dCBT-I)?

**Findings:**

In this cohort study of 4052 patients with insomnia, dCBT-I was superior to medication therapy at 6-month follow-up, although results were found to be unstable. The combination of dCBT-I and medication resulted in a sustained improvement in sleep quality compared with monotherapy modalities.

**Meaning:**

These findings suggest that dCBT-I needs to be thoughtfully combined with medication for long-term benefits in the treatment of insomnia and that further research into the design, implementation, and delivery of dCBT-I in terms of engagement and stability is required.

## Teaser

This cohort study evaluates the clinical effectiveness, engagement, durability, and adaptability of digital cognitive behavioral therapy for insomnia.

## Introduction

Insomnia disorder, comprising reports of poor sleep with associated daytime effects occurring 3 or more nights per week for 3 or more months,^1^ presents in 10% to 20% of adults worldwide.^2,3,4^ Compared with before the COVID-19 pandemic,^5,6,7^ the number of people experiencing insomnia is increasing rapidly, with the prevalence of clinically significant insomnia growing between 47% and 189%, leading to impaired work productivity and reduced quality of life.^8^ Insomnia is detrimental to human functions such as immunity and metabolism.^9^ It may exacerbate the cognitive and affective features of neurodegeneration and contribute to disease progression by accelerating pathogenic processes.^9^ In addition, insomnia has comorbid mental health disorders, such as depression and anxiety,^10,11^ and physical disorders, such as cardiovascular disease, type 2 diabetes, and increased fatigue.^12^ Therefore, it is urgent to identify an efficient intervention for insomnia with low-cost and wide dissemination.^13^

Digital cognitive behavioral therapy for insomnia (dCBT-I), by delivering the core content of cognitive behavioral therapy for insomnia (CBT-I) through the internet or mobile apps, includes 5 therapeutic sessions: stimulus control (SC), sleep restriction (SR), relaxation training (RT), cognitive reconstruction (CR), and sleep hygiene (SH).^14^ These 5 components can be used alone or in combination, and multicomponent cognitive behavioral therapy is strongly recommended in clinical practice guidelines.^15^ As a low-cost complement to CBT-I, dCBT-I has demonstrated efficacy in randomized clinical trials (RCTs) and meta-analyses for decreasing insomnia severity, enhancing sleep quality, reducing sleep medication intake, and mitigating comorbid mental disorders.^16,17,18,19,20^ It has been recommended as an effective and cost-saving alternative to sleeping pills by the National Institute for Health and Care Excellence of the US and England to reduce patients’ reliance on drugs, such as zolpidem and zopiclone, which can be dependence forming.^21^

However, existing studies exploring the efficacy of dCBT-I are mostly RCTs, mainly comparing the efficacy of dCBT-I with face-to-face CBT-I and usual care.^19,22,23,24,25,26,27^ There are also RCTs examining the efficacy of face-to-face CBT-I vs medication monotherapy and combination therapy.^28,29,30^ Despite the advantages of RCTs in removing the influence of external factors, they are cumbersome and time-consuming and often do not match complex clinical scenarios.^31^ Practice-based evidence of the effectiveness of dCBT-I can be acquired through analysis of clinical data.^32,33,34^ Notably, clinical data of health care delivery is routinely collected from a variety of settings, and the derived evidence can be more useful and generalizable than that from RCTs.^35^ Although there are also some retrospective studies exploring dCBT-I, such as SHUTi, Sleepio, and DREAM, these studies analyzed the effectiveness of dCBT-I primarily by comparing outcomes before and after the intervention, or by comparing it with patient education or routine care.^34,36,37,38,39,40^ To our knowledge, few retrospective studies on insomnia have been conducted to compare dCBT-I, medication therapy, and their combination. Furthermore, there is a lack of systematic analysis regarding dCBT-I engagement, durability, and adaptability in the practice setting, which is essential to inform effective and widespread dissemination. In other words, the potential of clinical data to explore the clinical effectiveness of dCBT-I has been severely underestimated.^41^

In this work, we conducted a retrospective cohort study (eFigure 1 in Supplement 1) using longitudinal data of the Chinese population collected from a mobile app called Good Sleep 365 (eFigure 2 in Supplement 1).^42^ Based on 6-month follow-up, the effectiveness of 3 treatment modalities, including dCBT-I, medication therapy, and their combination, was measured and compared. Retrospective studies are prone to bias in the selection of case and control sources and may introduce factors that confound the association between treatments and outcomes.^43,44^ To mitigate this problem, we first used inverse probability of treatment weighting (IPTW) to reduce the imbalance of measured confounders between treatment groups, which has already been proven effective in multiple clinical retrospective studies.^45,46,47,48^ In addition, patient engagement and dCBT-I effectiveness in different subpopulations were also systematically examined.

## Methods

### Ethical Declaration

All data used in this study were deidentified according to Health Insurance Portability and Accountability Act guidelines. The requirement for informed consent was waived because all available data were anonymous. The use of clinical information and data collection protocols were approved by the ethical committee of College of Biomedical Engineering and Instrument Science, Zhejiang University. This study followed the Strengthening the Reporting of Observational Studies in Epidemiology (STROBE) reporting guideline.

### Data Source

This retrospective study used data collected using Good Sleep 365 between November 14, 2018, and February 28, 2022. The app provided functions such as CBT-I training, medication records, and sleep quality assessment. Demographic characteristics (eg, sex, age) and clinical data related to insomnia (eg, family history of insomnia) were filled in at the time of enrollment. Therapeutic modes were allocated as prescribed by attending physicians (including 7 senior physicians with approximately 20 years of experience and 9 junior physicians with approximately 10 years of experience), and intervention details were documented in medication records, dCBT-I records, and sleep diaries. The participant’s treatment trajectory was remotely monitored through the app, and physicians regularly reminded the participant to complete the assessment scales, including PSQI, Epworth Sleepiness Sore (ESS), 7-item Generalized Anxiety Disorder (GAD-7), 9-item Patient Health Questionnaire (PHQ-9), and 15-item Patient Health Questionnaire (PHQ-15), to evaluate the treatment effectiveness. Overall, data available for analysis included cross-sectional data, such as patient demographic characteristics, insomnia-related clinical information, diagnoses, and complications, as well as longitudinal data documenting intervention details and assessment scores during follow-up.

### Study Design and Population

We conducted a retrospective cohort study to assess the clinical effectiveness, engagement, durability, and adaptability of dCBT-I, medication therapy, and their combination in the treatment of insomnia, using IPTW to adjust for confounding in intervention mode selection. Participants were allocated to 3 treatment groups according to prescriptions. Taking the first outpatient visit as the baseline, the improvement of sleep quality and complications within 6 months after the baseline was analyzed.

The source population consisted of all outpatients with the main concern of insomnia between November 14, 2018, to February 28, 2022. Following previous studies,^19,49^ we included patients (1) aged 18 to 80 years; (2) with no severe psychiatric disorders, such as restless legs syndrome, sleep apnea symptoms, epilepsy, or bipolar disorder; (3) with a PSQI score greater than 5 at baseline; (4) and with an ESS score of 10 or less at baseline. We excluded participants with incomplete demographic or treatment information. In addition, we retained participants who had completed at least 1 assessment during the 6-month follow-up period to ensure that the effectiveness of the intervention could be measured.

### Exposure, Covariates, and Outcomes

The primary exposure of interest was treatment with dCBT-I, medication therapy, or combination therapy. Patients in the dCBT-I and medication groups received the corresponding monotherapy, respectively. Patients in the combination group received the combined therapy of dCBT-I and hypnotic medications. Baseline (ie, at time of outpatient visit) covariates included demographic characteristics, insomnia-related clinical information, insomnia severity, and comorbidities. Both PSQI and its subitems (except part F) were used as the primary outcomes to assess sleep quality, and the secondary outcomes included the ESS, GAD-7, PHQ-9 and PHQ-15 to assess the severity of comorbid disorders. In this study, both the primary and secondary outcomes were assessed at month 1, month 3, and month 6 (primary end point). More details are in eAppendix 1 in Supplement 1.

### Statistical Analysis

We used IPTW to reduce imbalance in measured confounders between treatment groups, which has already been proved as an effective approach in multiple clinical retrospective studies (eMethods in Supplement 1).^45,46,47,48,50^ The propensity scores were based on the logistic regression models using multiple covariates as independent variables and each treatment group as the dependent variable. The mitigation of between-group differences by IPTW was fulfilled by using the R library ipw version 1.0-11. We calculated standardized mean differences (SMDs) for each covariate to determine whether there was significant difference between the 3 therapeutic groups. When the SMD was 0.1 or less after IPTW, the confounder was considered to have no between-group difference.^51,52^ Outcomes were comparable when there were no between-group differences in all covariates.

Proportions of categorical variables and mean/SD of numerical variables were calculated to represent the composition of the demographic and insomnia-related clinical data of patients. We primarily analyzed the effectiveness, durability, and patient engagement of 3 interventions for insomnia. Intervention effectiveness was evaluated based on response rates, changes in outcomes, and effect sizes. Then we analyzed 3 modes for comorbidities including somnolence, anxiety, depression, and somatic symptoms. Additionally, we performed a subgroup analysis, comparing the effectiveness of the 3 intervention modes in subpopulations with different characteristics. A strict *P* value threshold of .01 was used to handle the potential for type I error due to multiple comparisons. More details are in eAppendix 2 in Supplement 1. Statistical analysis was implemented in Python version 3.8.0 (Python Software Foundation) and R version 4.1.3 (R Project for Statistical Computing). Result plotting was with Prism version 9.4.0 (GraphPad) and R.

## Results

### Cohort Characteristics

**Figure 1.**
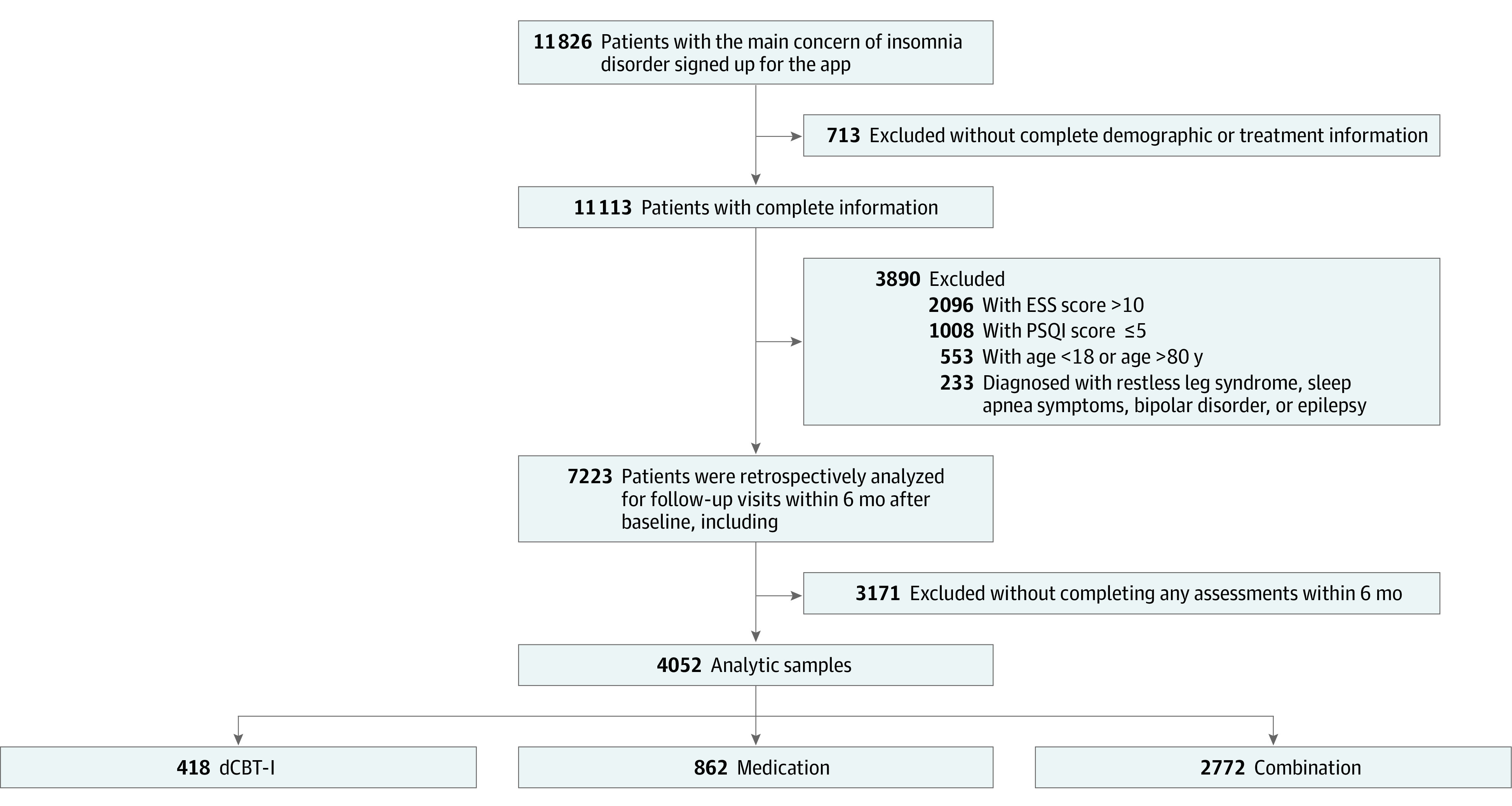
Cohort Selection Procedure dCBT-I indicates digital cognitive behavioral therapy for insomnia; ESS, Epworth Sleepiness Score; and PSQI, Pittsburgh Sleep Quality Index.

Between November 14, 2018, and February 28, 2022, 11 826 outpatients with the chief concern of insomnia disorder signed up for the app and obtained access. Patients used the app to receive dCBT-I, record their sleep and medication dairies, and fill out regular assessments for PSQI, ESS, GAD-7, PHQ-9, and PHQ-15. The detailed cohort selection procedure is illustrated in Figure 1. In total, 713 patients were excluded due to lack of complete demographic or treatment information; 786 patients were excluded due to inappropriate age or serious complications; 3104 patients with mild insomnia or somnolence at baseline were excluded; and 3171 patients were excluded without any outcome assessment within 6 months after baseline. As a result, the remaining 4052 eligible patients (34.26%) were included in the cohort, with a mean (SD) age of 44.29 (12.01) years and 3028 (74.7%) female participants. Specifically, there were 418 (10.32%), 862 (21.27%), and 2772 (68.41%) in the dCBT-I group, the medication group, and the combination group, respectively. The Table shows the baseline characteristics of this cohort. After IPTW adjustment, all baseline covariates met thresholds of SMD of 0.1 or less, indicating that between-group differences in covariates were eliminated and that differences in outcomes stemmed from different therapeutic modes (eTable 1 in Supplement 1).

**Table.** Demographic and Clinical Characteristics of the Cohort

Characteristic	Patients, No. (%)				*P* value
	Overall (N = 4052)	dCBT-I (n = 418)	Medication (n = 862)	Combination (n = 2772)	
Sex					
Male	1024 (25.3)	118 (28.2)	220 (25.5)	686 (24.7)	.31
Female	3028 (74.7)	300 (71.8)	642 (74.5)	2086 (75.3)	.31
Age, mean (SD), y	44.29 (12.01)	40.23 (11.73)	42.55 (13.69)	45.45 (11.27)	<.001
Education					
Primary	473 (11.7)	30 (7.2)	151 (17.5)	292 (10.5)	<.001
Middle and senior	1672 (41.3)	129 (30.9)	329 (38.2)	1214 (43.8)	<.001
College and undergraduate	1736 (42.8)	224 (53.6)	351 (40.7)	1161 (41.9)	<.001
Postgraduate	171 (4.2)	35 (8.4)	31 (3.6)	105 (3.8)	<.001
Employment					
Employed	3427 (84.6)	379 (90.7)	728 (84.5)	2320 (83.7)	.001
Unemployed	625 (15.4)	39 (9.3)	134 (15.5)	452 (16.3)	.001
Insomnia duration					
<1 mo	423 (10.4)	77 (18.4)	91 (10.6)	255 (9.2)	<.001
1-3 mos	573 (14.1)	69 (16.5)	124 (14.4)	380 (13.7)	.30
3-12 mos	771 (19.0)	61 (14.6)	192 (22.3)	518 (18.7)	.003
1-3 y	743 (18.3)	66 (15.8)	164 (19.0)	513 (18.5)	.34
3-5 y	487 (12.0)	52 (12.4)	111 (12.9)	324 (11.7)	.62
5-10 y	1055 (26.0)	93 (22.2)	180 (20.9)	782 (28.2)	<.001
Family history					
Yes	1298 (32.0)	145 (34.7)	246 (28.5)	907 (32.7)	.03
No	2754 (68.0)	273 (65.3)	616 (71.5)	1865 (67.3)	.03
First onset					
Yes	1004 (24.8)	99 (23.7)	215 (24.9)	690 (24.9)	.86
No	3048 (75.2)	319 (76.3)	647 (75.1)	2082 (75.1)	.86
Medication history					
Yes	2380 (58.7)	178 (42.6)	550 (63.8)	1652 (59.6)	<.001
No	1672 (41.3)	240 (57.4)	312 (36.2)	1120 (40.4)	<.001
Current medication					
Yes	2896 (71.5)	157 (37.6)	654 (75.9)	2085 (75.2)	<.001
No	1156 (28.5)	261 (62.4)	208 (24.1)	687 (24.8)	<.001
PSQI score at baseline, mean (SD)	14.99 (3.65)	12.60 (3.78)	15.09 (3.62)	15.32 (3.50)	<.001
ESS score, mean (SD)	3.45 (2.83)	4.21 (2.95)	3.50 (2.87)	3.32 (2.78)	<.001
GAD-7 score at baseline, mean (SD)	7.65 (5.83)	6.70 (5.49)	8.97 (6.23)	7.39 (5.69)	<.001
PHQ-9 score at baseline, mean (SD)	8.72 (6.19)	7.51 (5.64)	10.58 (7.01)	8.33 (5.87)	<.001
PHQ-15 score at baseline, mean (SD)	8.91 (4.58)	8.19 (4.59)	9.72 (4.78)	8.76 (4.49)	<.001

Abbreviations: dCBT-I, digital cognitive behavioral therapy for insomnia; ESS, Epworth Sleepiness Score; GAD-7, 7-item Generalized Anxiety Disorder; PHQ-9, 9-item Patient Health Questionnaire; PHQ-15, 15-item Patient Health Questionnaire; PSQI, Pittsburgh Sleep Quality Index.

### Response Rates

Overall, 243 of 317 (77.30%), 200 of 244 (81.97%), and 92 of 120 (76.19%) participants developed dCBT-I responses at 1-, 3- and 6-month follow-up (eTable 2 in Supplement 1). In contrast, 275 of 496 (55.45%), 251 of 453 (55.45%), and 132 of 245 (54.08%) participants responded to medication therapy and 1630 of 2418 (67.40%), 1653 of 2224 (74.34%), and 1152 of 1510 (76.31%) participants responded to combination therapy at 1-, 3-, and 6-month follow-up, respectively. The secondary outcomes also demonstrated significant benefits of dCBT-I and combination therapy (GAD-7: 76 of 120 [63.44%] and 1088 of 1511 [72.03%]; PHQ-9: 73 of 120 [60.69%] and 1086 of 1507 [72.08%]; and PHQ-15: 55 of 119 [46.34%] and 809 of 1482 [54.56%]) at 6-month follow-up. The detailed results are presented in eAppendix 3 in Supplement 1.

### Changes in Outcomes

Consistent findings were found on the basis of changes in primary and secondary outcomes. Improvements in PSQI with dCBT-I and combination therapy were observed from baseline to 6-month follow-up (from a mean [SD] of 13.51 [3.03] to 7.15 [3.25] for dCBT-I and from a mean [SD] of 12.92 [3.49] to 6.98 [3.43] for combination therapy), while pharmacological interventions (from a mean [SD] of 12.85 [3.49] to 8.92 [4.03]) were less effective (eTable 3 and eAppendix 3 in Supplement 1).

### Treatment Effect Sizes

**Figure 2.**
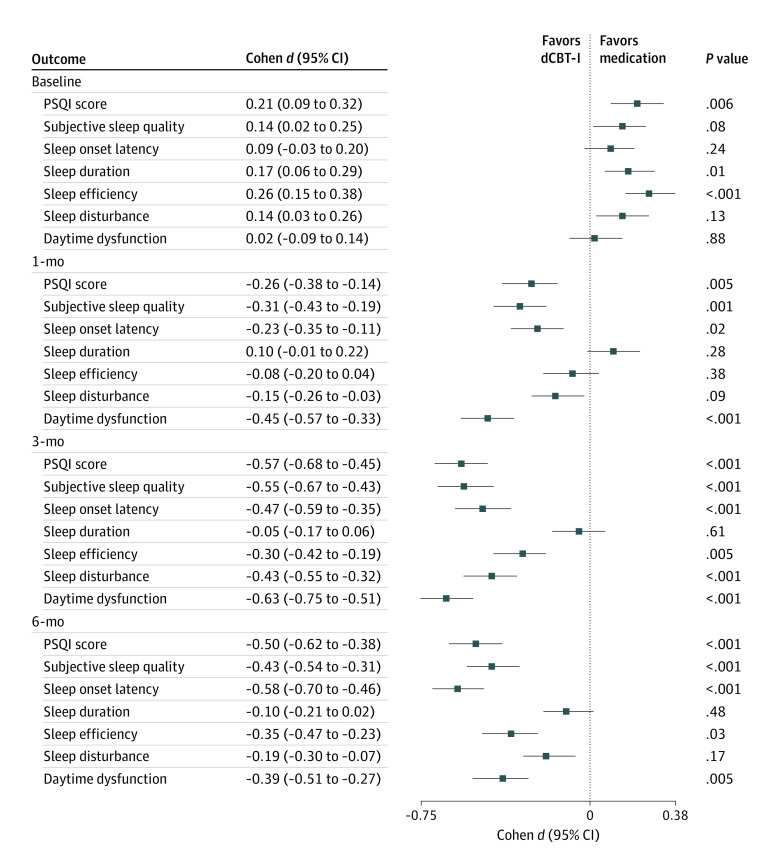
Forest Plots of Primary Outcomes of Digital Cognitive Behavioral Therapy for Insomnia (dCBT-I) vs Medication Therapy Each horizontal line represents the 95% CI of outcome comparison (Cohen *d* effect size), and the result is plotted as a box in the middle of the horizontal line. The vertical line is a reference line, placed at the value where there is no difference between 2 interventions. PSQI indicates Pittsburgh Sleep Quality Index.

**Figure 3.**
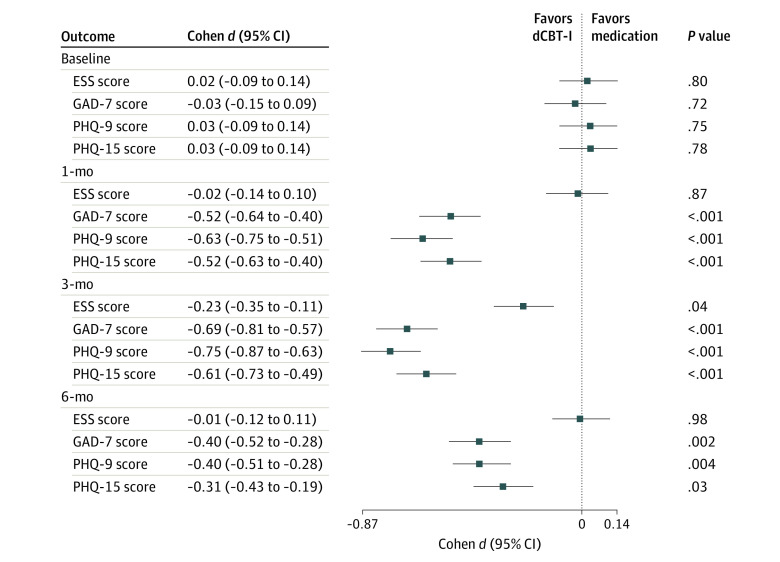
Forest Plots of Secondary Outcomes of Digital Cognitive Behavioral Therapy for Insomnia (dCBT-I) vs Medication Therapy Each horizontal line represents the 95% CI of outcome comparison (Cohen *d* effect size), and the results are plotted as a box in the middle of the horizontal line. The vertical line is a reference line, placed at the value where there is no difference between the 2 interventions. ESS indicates Epworth Sleepiness Score; GAD-7, 7-item Generalized Anxiety Disorder; PHQ-9, 9-item Patient Health Questionnaire; PHQ-15, 15-item Patient Health Questionnaire.

The comparison between dCBT-I and medication therapy showed that dCBT-I recipients had a more significant reduction in PSQI scores compared with the medication group at month 1 (small effect size: Cohen *d*, −0.26; 95% CI, −0.38 to −0.14; *P* = .005; SMD = 0.25), month 3 (moderate effect size: Cohen *d*, −0.57; 95% CI, −0.68 to −0.45; *P* < .001; SMD = 0.55), and month 6 (moderate effect size: Cohen *d*, −0.50; 95% CI, −0.62 to −0.38; *P* < .001; SMD = 0.484) (Figures 2 and 3). In particular, dCBT-I was superior to medication therapy in terms of subjective sleep quality, sleep onset latency, sleep efficiency, and daytime dysfunction at 6 months. Likewise, dCBT-I was more effective in improving all secondary outcomes. Taking depression as an example, dCBT-I produced a moderate effect size at month 1 (Cohen *d*, −0.63, 95% CI, −0.75 to −0.51; *P* < .001; SMD = 0.576); a moderate effect size at month 3 (Cohen *d*, −0.75; 95% CI, −0.87 to −0.63; *P* < .001; SMD = 0.667); and a small effect size at month 6 (Cohen *d*, −0.40; 95% CI, −0.51 to −0.28; *P* = .004; SMD = 0.381). Comparisons of primary and secondary outcomes across the 3 follow-up visits are presented in eTables 4 to 7 in Supplement 1. eFigures 3 and 4 in Supplement 1 show forest plots of medication vs combination therapy and dCBT-I vs combination therapy, respectively.

### Durability

The 3 interventions showed significant change in symptoms after 1 month of intervention and then gradually stabilized on all outcomes (eTable 8 and eFigure 5 in Supplement 1). The symptoms of patients receiving combination therapy steadily improved, with sustainable changes on all outcomes. After 3 months of intervention, the performance on the primary outcomes gradually stabilized within a certain range. In contrast, outcomes of dCBT-I improved steadily and rapidly during the first 3 months and then fluctuated. The change with respect to each outcome for patients receiving medication leveled off between month 1 and month 2, and even worsened after 4 to 5 months, indicating unstable durability. Combination therapy also had persistent and steady downward trends (good durability) in improving the secondary outcomes in comparison with dCBT-I, which had a rebound trend after 5 months, whereas medication monotherapy was slower to improve these comorbid disorders. Notably, the score of sleep medication use increased from baseline to the first month for both medication and combination therapies. Likewise, ESS score increased dramatically in dCBT-I and combination therapy at month 1.

### Engagement

Overall, 36.58% of patients participated in all components of dCBT-I. Almost all patients completed RT (98.40%) and SR (92.07%) during the 6-month period, while only 48.68% completed SC, and 44.33% completed CR. During the first month of treatment, 56.02%, 80.03%, and 94.14% of participants completed SH, SR, and RT, respectively, while only 8.78% and 8.93% participated in SC and CR (eFigure 6 and eTable 9 in Supplement 1). Since then, monthly engagements in SH (from 56.02% to 8.56%), SR (from 80.03% to 42.66%), and RT (from 94.14% to 49.91%) showed a downward trend, while participants in CR and SC increased gradually in the first 3 months. This observation is intuitive because in the dCBT-I program, the 5 sessions are organized in the order of SH, RT, SR, SC, and CR. Overall, patient adherence during the dCBT-I program (the first 3 months) was significantly better than after the program.

### Subgroup Analysis

**Figure 4.**
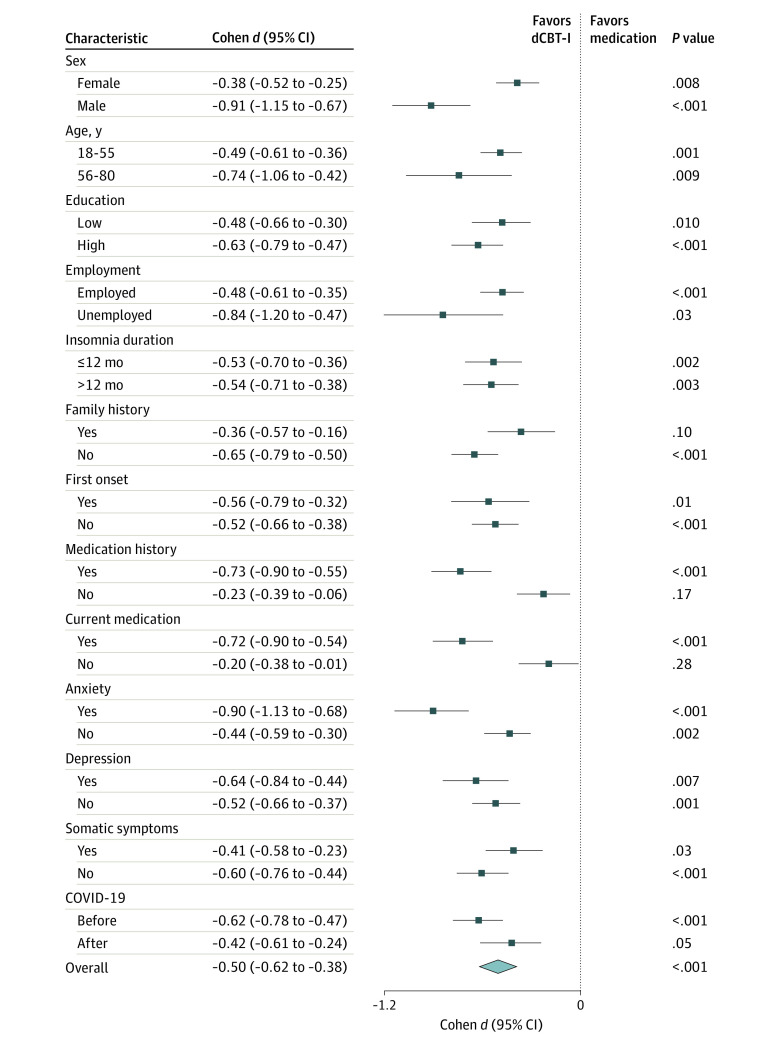
Subgroup Analysis Comparing the Effectiveness of Digital Cognitive Behavioral Therapy for Insomnia (dCBT-I) and Medication Therapy Each horizontal line represents the 95% CI of comparison (Cohen *d* effect size) of the Pittsburgh Sleep Quality Index score at month 6 in a subgroup, and the result is plotted as a box in the middle of the horizontal line. The last row indicates the comparison of effectiveness on the whole population. The vertical line is a reference line, placed at the value where there is no difference between 2 interventions. Education level and insomnia duration were coarsely categorized to avoid overclassification resulting in a smaller sample to influence confounders’ control with inverse probability of treatment weighting. Patients with primary, middle, and senior education were classified as having low education, while patients with higher education were classified as having high education. Insomnia duration refers to the length of interval from the first occurrence of insomnia to baseline. First onset indicates whether this insomnia was the first attack. Medication history indicates whether the patient had a history of taking any form of hypnotic medications prior to baseline. Current medication indicates whether the patient was taking any form of hypnotic medications at baseline.

Three therapeutic modes had varied superiority in distinct subgroups (Figure 4 and eFigures 7 and 8 in Supplement 1). Consistent with the primary analysis, dCBT-I and combination therapy showed a greater advantage than medication treatment in all subpopulations, albeit with variations across subgroups. The detailed results are presented in eAppendix 4 in Supplement 1.

## Discussion

Existing studies exploring the efficacy of dCBT-I are mostly RCTs, mainly comparing dCBT-I with face-to-face CBT-I and routine care.^22,23,36^ There are also RCTs examining the efficacy of face-to-face CBT-I vs medication monotherapy and combination therapy.^26,28,29^ Despite the advantages of RCTs in removing the influence of external factors, they are cumbersome and time-consuming and often do not match complex clinical scenarios.^31^ On the other hand, current retrospective studies primarily analyze the effectiveness of dCBT-I by comparing outcomes before and after the intervention or by comparing it with patient education or routine care.^34,36,37,38,39,40^ There is a lack of retrospective studies to compare the effectiveness of dCBT-I, medication, and their combination.^34,37,38,40^ Furthermore, few studies have explored the engagement, durability, and adaptability of dCBT-I, which has great potential to provide supportive evidence for clinically targeted therapy. To our knowledge, this study is the first to systematically explore dCBT-I from multiple perspectives based on clinical data. Overall, clinical evidence suggests that combination therapy outperformed on most of the primary (PSQI score and its subitems) and secondary (GAD-7 score, PHQ-9 score, and PHQ-15 score) outcomes, and patients in distinct subgroups had varied adaptability under the 3 therapeutic modes. Those receiving dCBT-I achieved statistically significant improvement in the primary and secondary outcomes in comparison with those receiving medication alone (Figures 2 and 3). Combination therapy showed better durability of outcome improvement than the other 2 therapeutic modes. Overall, our main findings about effectiveness are consistent with previous findings that CBT-I can achieve better sleep health outcomes compared with medication therapy,^25,26,53^ combinations of CBT-I and medications had the potential to optimize outcomes.^28,53,54,55^

Our results add to considerable clinical evidence for insomnia treatment. At 6-month follow up, insomnia improvements for those receiving dCBT-I were promising (eTables 2 and 3 in Supplement 1). The response rate of using dCBT-I in practice settings was comparable with that reported in previous RCTs (around 45%-60%).^56,57,58^ According to PSQI scores, 77.30%, 81.97%, 76.19% of patients receiving dCBT-I responded at 1-, 3-, and 6-month follow-up, respectively. dCBT-I was also more effective for comorbid disorders in comparison with medication, showing consistent superiority from the first month in relief of anxiety, depression, and somatic symptoms. Notably, the association of dCBT-I with reduction of ESS score started later than 1 month. One potential reason is that the abrupt implementation of the SR component in dCBT-I is associated with an increase in daytime somnolence, resulting in a sharp increase in ESS before stabilizing within reasonable limits.^59,60,61^ Moreover, dCBT-I was associated with greater reductions in ESS than the other modes, probably because taking sleep medication would cause the symptoms of hypersomnia.

Overall, patient engagement in dCBT-I was relatively low (36.58% completed all dCBT-I sessions), and the proportions of patients who attended the different sessions varied. Most of patients completed RT (98.40%) and SR (92.07%), while SC (48.68%), SH (68.71%), and CR (44.33%) had lower adherence (eTable 9 in Supplement 1). Interestingly, nearly 60% of patients did not complete 5 dCBT-I sessions but spontaneously selected several of them, reflecting problems with dCBT-I implementation in a clinical setting. dCBT-I is a digital channel to deliver criterion-standard treatment for insomnia, and its advantage of providing evidence-based insomnia treatment at scale has been widely recognized in RCT studies.^62,63^ However, it seems that populations are consistently less likely to initiate, continue, or complete all behavioral health treatments. Additionally, insignificant and unstable trends in outcome improvements with dCBT-I (ie, durability) were observed in the clinical data, indicating that the engagement challenge still exists when patients are asked to make major behavioral changes in their daily life. Many efforts have been spent on addressing this issue, while most research on dCBT-I and face-to-face CBT-I is based on therapist-guided interventions to improve patient engagement, albeit at a higher cost.^64,65^ To increase patient engagement with self-help dCBT-I, innovational therapeutic content should be developed and consistently updated to intrigue patients to overhaul their sleep patterns, behaviors, and thoughts.^66^ While existing studies show that culturally tailored health programming is associated with improved treatment outcomes,^67,68,69^ there have been limited efforts for Chinese individuals with insomnia. As it appears that tailored dCBT-I content may be important to maximizing the treatment effect, future studies in how to successfully adapt this treatment approach in the practice setting are needed.

In addition, patients within different subgroups showed different adaptability under the 3 therapeutic modes (Figure 4 and eFigures 7 and 8 in Supplement 1). Both dCBT-I and combination therapy have shown effectiveness advantages over medication monotherapy in all subpopulations, indicating the generalizability of both interventions. For patients with a history of taking hypnotics before intervention, the advantage of dCBT-I over medication therapy was more pronounced, showing a moderate effect size. One potential reason is that long-term use of hypnotics might lead to drug resistance, as confirmed in previous studies.^25,26^ Male patients gained more benefits with dCBT-I than female patients, possibly because female patients were prone to engage in unhelpful cognitive processes, such as rumination and worry, which made it more difficult to improve sleep quality.^70,71^ The obtained results of most subgroup analyses showed that dCBT-I and combination therapy could offer tangible benefits to patients with insomnia disorder. Notably, the COVID-19 epidemic appeared to have a negative association with the effectiveness of dCBT-I. dCBT-I produced a medium effect size compared with medication therapy before the pandemic. However, the effect size decreased after COVID-19. Combination treatment showed no significant difference in effectiveness vs dCBT-I before COVID-19 but outperformed with a small effect size afterward. The reason might be the poor adaptability of the current dCBT-I design to the pandemic or that the pandemic aggravated anxiety and made it difficult to achieve better effectiveness through dCBT-I monotherapy.^7,72^ dCBT-I should be optimized for use during pandemics for better insomnia management.^73,74^

### Limitations

This study has limitations. First, retrospective studies are not as standardized as prospective analyses owing to the nature of retrospective study design. Although common demographic and clinical factors that may potentially affect the outcomes were accounted for, and IPTW was used to balance between-group differences in this study, potential bias may be introduced from no blinding of participants and therapists or from any invisible confounder that may exist whose value cannot be well adjusted. Another limitation is the lack of study samples, which may explain the fluctuations in outcomes over time. We cannot draw firm conclusions about the drug resistance and instability of dCBT-I observed in this study. Further studies on a large scale of population with a large volume of clinical data are required. Third, patients with invisible outcomes were excluded from the study to ensure the accuracy of the effectiveness comparisons. However, bias may also be introduced. While retrospective studies have the potential to provide clinical evidence to evaluate the effectiveness of treatment modalities, reliable evidence stems from pragmatic RCTs with extensive inclusion criteria. In this sense, prospective studies are planned to further validate our findings.

## Conclusions

The results of this retrospective cohort study conducted in China suggest that dCBT-I is effective and has long-term benefits in patients with insomnia and that the combination of medication and dCBT-I is associated with sustained improvement in sleep quality compared with medication monotherapy. These positive findings provide clinical evidence that dCBT-I contributes to meaningful sleep improvements. Given the unstable durability of dCBT-I at 6-month follow-up, the design, implementation, and delivery of dCBT-I in the practice setting warrants further investigation.

## Notes

Supplement 1. **eAppendix 1.** Exposure, Covariates, and Outcomes **eAppendix 2.** Statistical Analysis **eAppendix 3.** Results of Response Rates and Changes in Outcomes **eAppendix 4.** Results of Subgroup Analysis **eMethods.** Confounders Control by IPTW **eTable 1.** Details of Covariates Before/After Adjustment by IPTW **eTable 2.** Response Rates According to Treatment Group **eTable 3.** Improvement of Primary and Secondary Outcomes According to Treatment Group **eTable 4.** Primary and Secondary Outcomes According to Treatment Group **eTable 5.** Comparison of Primary and Secondary Outcomes in the dCBT-I and Medication Groups **eTable 6.** Comparison of Primary and Secondary Outcomes in the Medication and Combination Groups **eTable 7.** Comparison of Primary and Secondary Outcomes in the dCBT-I and Combination Groups **eTable 8.** Time Series of Primary and Secondary Outcomes **eTable 9.** Patient Engagement in Each dCBT-I Session **eFigure 1.** Study Design **eFigure 2.** Interface of Good Sleep 365 App **eFigure 3.** Forest Plots of Primary and Secondary Outcomes for Medication Therapy vs Combination Therapy **eFigure 4.** Forest Plots of Primary and Secondary Outcomes for dCBT-I vs Combination Therapy **eFigure 5.** Line Charts of Time Series of the Primary and Secondary Outcomes During a 6-Month Follow-up **eFigure 6.** Patient Engagement in Each of the 5 dCBT-I Sessions **eFigure 7.** Subgroup Analysis Comparing the Effectiveness of Medication Therapy and Combination Therapy **eFigure 8.** Subgroup Analysis Comparing the Effectiveness of dCBT-I and Combination Therapy
eReferences.

Supplement 2.
Data Sharing Statement
