# Supplementary material for: Comparative Effectiveness of Digital Cognitive Behavioral Therapy vs Medication Therapy Among Patients With Insomnia
Source: JAMA Netw Open. 2023 Apr 11;6(4):e237597. doi: 10.1001/jamanetworkopen.2023.7597 (PMC10091171; doi:10.1001/jamanetworkopen.2023.7597)
Supplement: Supplement 1. — eAppendix 1. Exposure, Covariates, and Outcomes eAppendix 2. Statistical Analysis eAppendix 3. Results of Response Rates and Changes in Outcomes eAppendix 4. Results of Subgroup Analysis eMethods. Confounders Control by IPTW eTable 1. Details of Covariates Before/After Adjustment by IPTW eTable 2. Response Rates According to Treatment Group eTable 3. Improvement of Primary and Secondary Outcomes According to Treatment Group eTable 4. Primary and Secondary Outcomes According to Treatment Group eTable 5. Comparison of Primary and Secondary Outcomes in the dCBT-I and Medication Groups eTable 6. Comparison of Primary and Secondary Outcomes in the Medication and Combination Groups eTable 7. Comparison of Primary and Secondary Outcomes in the dCBT-I and Combination Groups eTable 8. Time Series of Primary and Secondary Outcomes eTable 9. Patient Engagement in Each dCBT-I Session eFigure 1. Study Design eFigure 2. Interface of Good Sleep 365 App eFigure 3. Forest Plots of Primary and Secondary Outcomes for Medication Therapy vs Combination Therapy eFigure 4. Forest Plots of Primary and Secondary Outcomes for dCBT-I vs Combination Therapy eFigure 5. Line Charts of Time Series of the Primary and Secondary Outcomes During a 6-Month Follow-up eFigure 6. Patient Engagement in Each of the 5 dCBT-I Sessions eFigure 7. Subgroup Analysis Comparing the Effectiveness of Medication Therapy and Combination Therapy eFigure 8. Subgroup Analysis Comparing the Effectiveness of dCBT-I and Combination Therapy eReferences. [file jamanetwopen-e237597-s001.pdf]

Supplemental Online Content

Lu M, Zhang Y, Zhang J, et al. Comparative effectiveness of digital cognitive behavioral therapy vs medication therapy among patients with insomnia. *JAMA Netw Open.* 2023;6(4):e237597. doi:10.1001/jamanetworkopen.2023.7597

eAppendix 1. Exposure, Covariates, and Outcomes

eAppendix 2. Statistical Analysis

eAppendix 3. Results of Response Rates and Changes in Outcomes

eAppendix 4. Results of Subgroup Analysis

eMethods. Confounders Control by IPTW

eTable 1. Details of Covariates Before/After Adjustment by IPTW

eTable 2. Response Rates According to Treatment Group

eTable 3. Improvement of Primary and Secondary Outcomes According to Treatment Group

eTable 4. Primary and Secondary Outcomes According to Treatment Group

eTable 5. Comparison of Primary and Secondary Outcomes in the dCBT-I and Medication Groups

eTable 6. Comparison of Primary and Secondary Outcomes in the Medication and Combination Groups

eTable 7. Comparison of Primary and Secondary Outcomes in the dCBT-I and Combination Groups

eTable 8. Time Series of Primary and Secondary Outcomes

eTable 9. Patient Engagement in Each dCBT-I Session

eFigure 1. Study Design

eFigure 2. Interface of Good Sleep 365 App

eFigure 3. Forest Plots of Primary and Secondary Outcomes for Medication Therapy vs Combination Therapy

eFigure 4. Forest Plots of Primary and Secondary Outcomes for dCBT-I vs Combination Therapy

eFigure 5. Line Charts of Time Series of the Primary and Secondary Outcomes During a 6-Month Follow-up

eFigure 6. Patient Engagement in Each of the 5 dCBT-I Sessions

eFigure 7. Subgroup Analysis Comparing the Effectiveness of Medication Therapy and Combination Therapy

eFigure 8. Subgroup Analysis Comparing the Effectiveness of dCBT-I and Combination Therapy

eReferences.

This supplemental material has been provided by the authors to give readers additional information about their work.

© 2023 Lu M et al. *JAMA Network Open.*

eAppendix 1. Exposure, Covariates, and Outcomes.

Exposure: The primary exposure of interest was treatment with dCBT-I, medication therapy, or combination therapy. Three treatment groups were divided based on the prescriptions given by physicians. Patients in the dCBT-I and medication groups received the corresponding monotherapy, respectively. Patients in the combination group received the combined therapy of dCBT-I and hypnotic medications. dCBT-I treatment was performed through the App, which provides an automated, interactive, self-guided digital medium for adopting CBT-I in the real world. Courses were systematically tailored by physicians according to clinical guidelines,[1,2] which were divided into dCBT-I sessions as a 3-month program. First, an overview video was provided to introduce the preliminary knowledge of dCBT-I. For each dCBT-I session, instructions were provided to help recipients understand it smoothly. The content of each course was presented in the form of video or audio and was approximately 30-50 minutes long. Although sequential completion of all dCBT-I courses was encouraged, recipients had the flexibility to choose the courses they were interested in. Recipients could also revisit the courses in the App. Prescribed medications included multiple types of sleeping pills, such as benzodiazepines (i.e., lorazepam), non-benzodiazepines (i.e., eszopiclone) and the antidepressant trazodone. The most commonly used medications are shown in the table below. Patients receiving medication therapy and combination therapy could check their prescriptions and fill in their medication-taking behaviors in medication records of the App.

No.	Name	Per dose	Time
1	Zopiclone	7.5mg	before bedtime
2	Lorazepam	0.5mg-1mg	before bedtime
3	Trazodone	50mg-100mg	before bedtime
4	Quetiapine	50mg-100mg	before bedtime
5	Zolpidem Tartrate	5mg-10mg	before bedtime
6	Alprazolam	0.4mg-0.8mg	before bedtime
7	Mirtazapine	7.5mg-15mg	before bedtime

Covariates: We collected baseline (the outpatient visit time) covariates, including (1) demographic characteristics (sex, age, education experience, employment status); (2) insomnia-related clinical information (insomnia duration by the item of questionnaire "How long have you had insomnia symptoms?", current medication by the item of questionnaire "Are you currently taking hypnotics?", medication history by the item of questionnaire "Have you ever taken hypnotics?", first onset of insomnia by the item of questionnaire "Are you experiencing insomnia symptoms for the first time?", family history of insomnia); (3) insomnia severity (PSQI score at baseline); (4) comorbidities (somnolence measured by ESS score at baseline, anxiety measured by GAD-7 score at baseline, depression measured by PHQ-9 score at baseline, and somatic symptoms measured by PHQ-15 score at baseline). Of note, the covariate of current medication records whether patients take hypnotics at baseline (at the time of the first visit), while the covariate of medication history records whether patients have a history of taking hypnotics.

Outcomes: PSQI score is a comprehensive scale (range 0-21, with higher scores indicating poorer sleep quality and more severe insomnia. PSQI score > 5 denotes poor sleep quality and PSQI score $\leqslant$ 5 indicates no obvious insomnia [3]) for assessing sleep quality of patients. PSQI includes 7 subitems, namely subjective sleep quality (PSQI part A), sleep onset latency (PSQI part B, i.e., how long it takes to fall asleep), sleep duration (PSQI part C), sleep efficiency (PSQI part D, i.e., the percentage of time a person spends in bed), sleep disturbance (PSQI part E), sleep medication use (PSQI part F), and daytime dysfunction (PSQI part G), with 3 points for each subitem. Notably, part F was excluded in this study to avoid potential bias in treatment effect estimation, given that dCBT-I recipients barely take hypnotics. Both PSQI and its subitems (w/o part F) were employed as the primary outcomes.

Furthermore, to evaluate the treatment effect of distinct therapeutic modes on comorbid disorders, we took ESS, GAD-7, PHQ-9 and PHQ-15 as the secondary outcomes to assess the severity of somnolence, anxiety, depression, and somatic symptoms, respectively. In this study, both the primary and secondary outcomes were assessed at month 1, month 3, and month 6 (primary

© 2023 Lu M et al. *JAMA Network Open.*

endpoint), respectively. Because patients in the real world may continue to complete CBT-I courses or take hypnotic drugs after the 3-month course of treatment unlike RCTs, the outcomes at month 6 are defined as the long-term treatment outcomes, which indicates the outcomes of patients with continuous intervention.

eAppendix 2. Statistical Analysis.
Retrospective studies are prone to bias with respect to selection of sources of cases and controls, and inevitably exist factors that confound the relationship between treatment and outcome. To mitigate this problem, we employed IPTW (eMethod 1 in the Supplement) to reduce imbalance in measured confounders between treatment groups, which has already been proved as an effective approach in multiple clinical retrospective studies. [4–8] The propensity scores were based on the logistic regression models using multiple covariates (ref Section Covariates) as independent variables and each treatment group as the dependent variable. The mitigation of between-group differences by IPTW were fulfilled by using the R library ipw (versions 1.0-11). We calculated standardized mean differences (SMDs) for each covariate to determine whether there was significant difference of this confounder between the three therapeutic groups. When SMD≤0.1 for a confounder after IPTW, the confounder was considered to have no between-group difference.[9,10] Outcomes were comparable when there were no between-group differences in all covariates.

Proportions of categorical variables and mean/standard deviation (SD) of numerical variables were calculated to represent the composition of the demographic and insomnia-related clinical data of patients. We primarily analyzed the effectiveness, durability, and patient engagement of three interventions for insomnia. Intervention effectiveness was evaluated based on response rates, changes in outcomes, and effect sizes. Then we analyzed three modes for comorbidities including somnolence, anxiety, depression, and somatic symptoms. Besides, we performed a subgroup analysis, comparing the effectiveness of the three intervention modes in sub-populations with different characteristics. A strict p-value threshold of .01 was used to handle the potential for type I error due to multiple comparisons.

To facilitate clinical interpretation of our findings, we calculated response rates of the primary and secondary outcomes. Treatment response was defined as a change in PSQI score of ≥ 3 points. [11–13] Clinically significant improvement in PSQI components was defined as a ≥ 1 point change in PSQI component score. Treatment response for comorbidities was defined as a 50% decrease in pretreatment scores including ESS, GAD-7, PHQ-9, and PHQ-15 scores.[14] Chi-square tests (categorical data) or Kruskal-Wallis test (numerical data) were used to assess differences between the three groups.

In addition, changes in outcomes compared to the baseline were counted after 1, 3, and 6 months to characterize the effectiveness of three therapeutic modes. Cohen's d effect size was calculated as the adjusted mean difference divided by the sample SD and presented as a forest plot to estimate the treatment effect for each outcome between the two intervention modes. A positive Cohen's d value indicates that the second intervention is better than the first, and vice versa. Cutoff values for small, medium, and large effect sizes were defined as 0.2, 0.5, and 0.8, respectively.[15,16]

To assess the onset, effectiveness, and durability of each specific intervention mode on target outcomes, monthly averages from baseline to 6-month follow-up were calculated to form a time series, displayed as a line graph. To analyze patient engagement in self-guided dCBT-I, the proportion of patients who completed each dCBT-I session was measured at 6-month follow-up.

Subgroup analysis was performed in sub-populations with different demographics, clinical characteristics, and comorbidities, including anxiety (GAD-7 ≥ 10 at baseline), depression (PHQ-9 ≥ 10 at baseline), and somatic symptoms (PHQ-15 ≥ 10 at baseline). The effectiveness of three interventions on patients before and after COVID-19 was also evaluated, with January 1st, 2020 as the time cut-off. Consistent with the primary analysis, Cohen's d effect size and p-value were calculated to evaluate the therapeutic effectiveness of each intervention mode (denoted by PSQI score at month 6) in a subgroup. Statistical analysis was implemented in Python (version 3.8.0) and R (version 4.1.3). Result plotting was done by using the GraphPad Prism and R. eFigure 1 shows the research framework of this study.

© 2023 Lu M et al. *JAMA Network Open.*

eAppendix 3. Results of Response Rates and Changes in Outcomes.

Response Rates: According to changes in PSQI scores, 77.30%, 81.97%, and 76.19% of participants developed dCBT-I responses at 1-, 3- and 6-month follow-up (eTable 2). In contrast, 55.45%, 55.45%, 54.08% and 67.40%, 74.34%, 76.31% of the participants responded to medication therapy and combination therapy. Compared to combination therapy, dCBT-I achieved a greater proportion of responders on all primary outcomes at month 3, despite its superiority diminished at month 6. In addition, the response rate for almost all outcomes of combination therapy was significantly improved at month 6 compared with month 1 (taking the PSQI score as an example, the response rate of combination therapy improved from 67.40% to 76.31%). Conversely, several outcomes in the dCBT-I group showed a rebound trend from the 3rd month to the 6th month (taking the PSQI score as an example, the response rate of dCBT-I decreased from 81.97% to 76.19%). In addition, sleep disturbance (dCBT-I 45.30%, medication therapy 29.22%, combination therapy 41.01%) had the lowest response rate, indicating that they were relatively difficult to improve. The secondary outcomes also demonstrated significant benefits of dCBT-I and combination therapy, regarding the response rates of GAD-7 (63.44%, 72.03%), PHQ-9 (60.69%, 72.08%), and PHQ-15 (46.34%, 54.56%) at 6-month follow-up.

Changes in Outcomes: Three therapeutic modes showed reductions in all outcomes (except for ESS score) at month 1, month 3, and month 6 in comparison with baseline (eTable 3). Patients treated with dCBT-I (reducing PSQI score from 13.51 to 7.15) and combination therapy (from 12.92 to 6.98) showed more improvement during the 6-month follow-up, while pharmacological interventions (from 12.85 to 8.92) were less effective. Marginal advantages of dCBT-I were observed compared with combination therapy. Similar findings were observed in all sub-items of the PSQI score, with medication therapy showing the worst effectiveness. All three interventions resulted in a marginal increase in ESS scores. In addition, combination therapy was more effective than dCBT-I in the treatment of comorbidities including anxiety (decreased by 5.67 points with combination therapy versus decreased by 4.93 points with dCBT-I), depression (6.08 versus 5.49), and somatic symptoms (4.40 versus 3.78) during the 6-month follow-up.

eAppendix 4. Results of Subgroup Analysis.

Three therapeutic modes had varied superiority in distinct subgroups (Figure 4 in the main manuscript, eFigures 7 and 8). Consistent with the primary analysis, dCBT-I and combination therapy showed a greater advantage over medication treatment in all sub-populations, albeit with variations across subgroups. Specifically, dCBT-I had moderate effect sizes compared to medication therapy in most sub-populations. Notably, large effect sizes were shown in male patients (Cohen's d, -0.91, 95% CI, -1.15 to -0.67, p<.001), unemployed patients (-0.84, -1.20 to -0.47, p=.03), and patients with co-occurring anxiety (-0.90, -1.13 to -0.68, p<.001). However, no significant difference was found in patients who did not take hypnotic drugs before intervention (Medication history - No, -0.23, -0.39 to -0.06, p=.17). Likewise, combination therapy produced a large effect size in male patients (0.82, 0.67 to 0.98, p<.001) compared to medication therapy. There was no significant difference in effectiveness between dCBT-I and combination therapy in most subgroups. For patients who did not take hypnotic medication before baseline (Medication history - No, 0.26, 0.12 to 0.40, p=.05), combining dCBT-I with medication was more effective than dCBT-I monotherapy. For elderly (-0.40, -0.68 to -0.11, p=.07) and unemployed patients (-0.58, -0.91 to -0.26, p=.07), dCBT-I produced small and moderate effect sizes compared to the combination treatment, respectively.

The COVID-19 outbreak did not affect the significant advantages of dCBT-I and combination therapy over medication treatment. However, dCBT-I monotherapy did not produce as large an effect size after the outbreak (-0.42, -0.61 to -0.24, p=.05) as it did before the outbreak (-0.62, -0.78 to -0.47, p<.001), and the combination of dCBT-I and medication showed a slightly improved effect in comparison with dCBT-I after the outbreak (0.24, 0.07 to 0.41, p=.22).

eMethod 1. Confounders Control by IPTW.

This retrospective cohort study employed a propensity score framework to learn treatment assignment (dCBT-I, medication therapy, and combination therapy) and to control confounders among three groups. A propensity score-weighted method called

IPTW was applied to alleviate the problem of sample loss caused by traditional propensity score matching methods.

A triplet (X, G, Y) was used to represent real-world data of patients in the dCBT-I group, medication group, and combination group, where X, G, Y stand for the covariates, treatment assignment, and outcomes, respectively. The propensity score is defined as $P(G = 1|X)$, where P also represents the probability that X was assigned to the treatment group. The inverse of the probability (propensity score) was allocated to each patient as weights (ω), which can be represented as:

$$\omega = \begin{cases} \frac{1}{P(G = 1|X)}, \text{for treatment group} \\ \frac{1}{1 - P(G = 1|X)}, \text{for control group} \end{cases}$$

Thereafter, the integrating weight that IPTW assigns to each sample in either the treatment group or control group can be defined as:

$$\omega = \frac{G}{P(G = 1|X)} + \frac{1 - G}{1 - P(G = 1|X)}$$

Classification models including machine learning or deep learning models have the feasibility of obtaining propensity scores, with covariates as features and treatment assignments as labels. A logistic regression model was chosen in this study, with strong interpretability and wide application. Taking the X as the independent variable and G as the dependent variable, a multinomial logistic regression model was constructed to fit the data linearly. The predicted probability of each sample is considered as the propensity score, and the weight value of the sample can also be calculated according to the above formula. The weighted covariates and outcomes of patients were utilized for further analysis.

The performance of confounders control by IPTW was estimated by the goodness of balance, measured by the SMD between three cohorts. SMD is a measure of distance between two group means in terms of one or more variables. As it is standardized, comparison across variables on different scales is possible. SMD is defined as:

$$\text{SMD}(X_{treat}, X_{control}) = \frac{|\bar{X}_{treat} - \bar{X}_{control}|}{\sqrt{\frac{s_{treat}{}^2 + s_{control}{}^2}{2}}}$$

where X_{treat}, $X_{control}$ are the representations of covariates in the treated group and control group. $\bar{X}_{treat}$, $\bar{X}_{control}$ are their sample means and $s_{treat}{}^2$, $s_{control}{}^2$ are their sample variances over the treated group and control group, respectively. If SMD in a covariate is no greater than 0.1, it is considered to be balanced.

Visible covariates (X) in this study included sex (Female, Male), age, education experience (Primary education, Middle & Senior education, College & Undergraduate, Postgraduate), employment status (Employed, Unemployed), insomnia duration (<1 month, 1-3 months, 3-12 months, 1-3 years, 3-5 years, 5-10 years), current medication (Yes, No), medication history (Yes, No), first onset (Yes, No), family history (Yes, No), PSQI score at baseline, ESS score at baseline, GAD-7 score at baseline, PHQ-9 score at baseline, and PHQ-15 score at baseline. Treatment assignments (G) included dCBT-I, medication therapy, and combination therapy. Outcomes (Y) included primary outcomes (PSQI score, subjective sleep quality, sleep onset latency, sleep duration, sleep efficiency, sleep disturbance, and daytime dysfunction) and secondary outcomes (ESS score, GAD-7 score, PHQ-9 score, and PHQ-15 score). Categorical confounders were discretized based on one-hot encoding, and continuous variables were normalized to avoid the effect of magnitude on model fit. As there were three cohorts in this study, each group can be regarded as the treatment group. By serving one of three cohorts as the treatment one and the other two as the control group, three logistic regression models were fitted to obtain the corresponding probability as propensity score, and the inverse of propensity score was used as the weight for each patient sample. Three SMD values between any two of three groups were calculated and the mean of them was used to assess whether the confounder was balanced globally. If the mean SMD values of all covariates were no greater than 0.1, the confounders were considered to be controlled by IPTW and the outcomes were comparable.

© 2023 Lu M et al. *JAMA Network Open.*

eTable 1. The Details of Covariates Before/After Adjustment by IPTW.

	Before IPTW					After IPTW				
**Covariate**	**dCBT-I**	**Medication**	**Combination**	**p-value**	**SMD**	**dCBT-I**	**Medication**	**Combination**	**p-value**	**SMD**
Sex (%)										
Male	118 (28.2)	220 (25.5)	686 (24.7)	.31	0.053	89.1 (21.1)	224.3 (25.8)	695.4 (25.1)	.28	0.074
Female	300 (71.8)	642 (74.5)	2086 (75.3)	.31	0.053	332.8 (78.9)	644.3 (74.2)	2071.7 (74.9)	.28	0.074
Age, [year (mean (SD)]	40.23 (11.73)	42.55 (13.69)	45.45 (11.27)	<.001	0.289	44.72 (12.01)	44.54 (13.44)	44.42 (11.42)	.93	0.017
Education (%)										
Primary	30 (7.2)	151 (17.5)	292 (10.5)	<.001	0.213	49.5 (11.7)	105.5 (12.2)	331.3 (12.0)	.97	0.009
Middle & Senior	129 (30.9)	329 (38.2)	1214 (43.8)	<.001	0.18	179.1 (42.4)	363.4 (41.8)	1142.9 (41.3)	.92	0.015
College & Undergraduate	224 (53.6)	351 (40.7)	1161 (41.9)	<.001	0.173	177.5 (42.1)	362.7 (41.8)	1179.8 (42.6)	.92	0.012
Postgraduate	35 (8.4)	31 (3.6)	105 (3.8)	<.001	0.135	15.9 (3.8)	37.0 (4.3)	113.1 (4.1)	.9	0.017
Employment (%)										
Employed	379 (90.7)	728 (84.5)	2320 (83.7)	.001	0.14	347.1 (82.3)	730.9 (84.1)	2335.3 (84.4)	.72	0.038
Unemployed	39 (9.3)	134 (15.5)	452 (16.3)	.001	0.14	74.8 (17.7)	137.7 (15.9)	431.8 (15.6)	.72	0.038
Insomnia duration (%)										
<1 month	77 (18.4)	91 (10.6)	255 (9.2)	<.001	0.18	44.3 (10.5)	96.4 (11.1)	289.4 (10.5)	.89	0.014
1-3 months	69 (16.5)	124 (14.4)	380 (13.7)	.30	0.052	65.5 (15.5)	119.6 (13.8)	388.6 (14.0)	.76	0.033
3-12 months	61 (14.6)	192 (22.3)	518 (18.7)	.003	0.133	73.6 (17.4)	163.5 (18.8)	526.5 (19.0)	.81	0.027
1-3 years	66 (15.8)	164 (19.0)	513 (18.5)	.34	0.057	66.4 (15.7)	153.2 (17.6)	507.5 (18.3)	.58	0.046
3-5 years	52 (12.4)	111 (12.9)	324 (11.7)	.62	0.024	63.8 (15.1)	110.0 (12.7)	334.7 (12.1)	.42	0.059
5-10 years	93 (22.2)	180 (20.9)	782 (28.2)	<.001	0.114	108.4 (25.7)	226.0 (26.0)	720.4 (26.0)	.99	0.005
Family history (%)										
Yes	145 (34.7)	246 (28.5)	907 (32.7)	.03	0.088	150.2 (35.6)	271.6 (31.3)	886.8 (32.0)	.48	0.061
No	273 (65.3)	616 (71.5)	1865 (67.3)	.03	0.088	271.7 (64.4)	597.0 (68.7)	1880.3 (68.0)	.48	0.061
First onset (%)										
Yes	99 (23.7)	215 (24.9)	690 (24.9)	.86	0.02	94.2 (22.3)	222.6 (25.6)	685.5 (24.8)	.59	0.051
No	319 (76.3)	647 (75.1)	2082 (75.1)	.86	0.02	327.7 (77.7)	646.0 (74.4)	2081.7 (75.2)	.59	0.051
Medication history (%)										
Yes	178 (42.6)	550 (63.8)	1652 (59.6)	<.001	0.289	254.1 (60.2)	499.5 (57.5)	1630.8 (58.9)	.73	0.037

© 2023 Lu M et al. *JAMA Network Open.*

No	240 (57.4)	312 (36.2)	1120 (40.4)	<.001	0.289	167.9 (39.8)	369.1 (42.5)	1136.3 (41.1)	.73	0.037
Current medication (%)										
Yes	157 (37.6)	654 (75.9)	2085 (75.2)	<.001	0.558	300.6 (71.2)	610.7 (70.3)	1977.7 (71.5)	.85	0.017
No	261 (62.4)	208 (24.1)	687 (24.8)	<.001	0.558	121.4 (28.8)	257.9 (29.7)	789.4 (28.5)	.85	0.017
PSQI score at baseline (mean (SD))	12.60 (3.78)	15.09 (3.62)	15.32 (3.50)	<.001	0.495	15.02 (3.68)	14.96 (3.65)	15.00 (3.65)	.97	0.011
ESS score (mean (SD))	4.21 (2.95)	3.50 (2.87)	3.32 (2.78)	<.001	0.206	3.43 (2.81)	3.37 (2.79)	3.43 (2.82)	.88	0.014
GAD-7 score at baseline (mean (SD))	6.70 (5.49)	8.97 (6.23)	7.39 (5.69)	<.001	0.258	7.48 (5.88)	7.66 (5.86)	7.66 (5.82)	.93	0.021
PHQ-9 score at baseline (mean (SD))	7.51 (5.64)	10.58 (7.01)	8.33 (5.87)	<.001	0.324	8.79 (6.45)	8.61 (6.28)	8.72 (6.17)	.89	0.019
PHQ-15 score at baseline (mean (SD))	8.19 (4.59)	9.72 (4.78)	8.76 (4.49)	<.001	0.219	9.04 (4.90)	8.92 (4.52)	8.92 (4.59)	.96	0.018

p-value less (larger) than 0.05 and SMD larger (less) than 0.1 are denoted as red (blue) color.

© 2023 Lu M et al. *JAMA Network Open.*

eTable 2. Response Rates According to Treatment Group

	1-month	3-month	6-month
PSQI score			
dCBT-I	77.30%	81.97%	76.19%
Medication	55.45%	55.45%	54.08%
Combination	67.40%	74.34%	76.31%
Subjective sleep quality			
dCBT-I	73.83%	82.07%	71.86%
Medication	59.61%	58.43%	59.18%
Combination	68.86%	72.84%	71.96%
Sleep onset latency			
dCBT-I	62.14%	72.32%	71.59%
Medication	51.34%	52.97%	51.20%
Combination	65.21%	69.68%	71.05%
Sleep duration			
dCBT-I	42.29%	56.55%	58.13%
Medication	42.07%	44.91%	41.35%
Combination	43.33%	51.07%	52.05%
Sleep efficiency			
dCBT-I	50.76%	64.43%	61.51%
Medication	42.32%	41.85%	44.60%
Combination	51.63%	56.85%	60.49%
Sleep disturbance			
dCBT-I	36.43%	48.49%	45.30%
Medication	31.74%	30.62%	29.22%
Combination	33.10%	36.86%	41.01%
Daytime dysfunction			
dCBT-I	56.47%	64.09%	55.35%
Medication	38.75%	45.26%	44.53%
Combination	43.18%	57.00%	62.95%
ESS score			
dCBT-I	23.30%	25.09%	20.73%
Medication	25.39%	21.45%	22.44%
Combination	19.33%	23.18%	25.68%
GAD-7 score			
dCBT-I	60.93%	63.71%	63.44%
Medication	41.18%	47.45%	51.14%
Combination	57.04%	65.79%	72.03%
PHQ-9 score			
dCBT-I	64.50%	71.13%	60.69%
Medication	41.38%	43.94%	49.26%
Combination	54.98%	67.21%	72.08%
PHQ-15 score			
dCBT-I	45.32%	45.56%	46.34%

© 2023 Lu M et al. *JAMA Network Open.*

Medication	28.24%	31.67%	33.85%
Combination	32.93%	46.34%	54.56%

eTable 3. Improvement of Primary and Secondary Outcomes According to Treatment Group

Outcome	dCBT-I	Medication	Combination
PSQI score			
baseline	13.51	12.85	12.92
1-month	8.31 (-5.20)	9.27 (-3.58)	8.21 (-4.71)
3-month	7.3 (-6.21)	9.3 (-3.55)	7.29 (-5.63)
6-month	7.15 (-6.36)	8.92 (-3.93)	6.98 (-5.94)
Subjective sleep quality			
baseline	2.42	2.32	2.34
1-month	1.18 (-1.24)	1.44 (-0.88)	1.19 (-1.15)
3-month	1.01 (-1.41)	1.44 (-0.88)	1.06 (-1.28)
6-month	1.15 (-1.27)	1.47 (-0.85)	1.04 (-1.30)
Sleep onset latency			
baseline	2.47	2.40	2.37
1-month	1.45 (-1.02)	1.66 (-0.74)	1.26 (-1.11)
3-month	1.28 (-1.19)	1.68 (-0.72)	1.15 (-1.22)
6-month	1.23 (-1.24)	1.72 (-0.68)	1.12 (-1.25)
Sleep duration			
baseline	2.50	2.33	2.37
1-month	1.77 (-0.73)	1.65 (-0.68)	1.71 (-0.66)
3-month	1.62 (-0.88)	1.68 (-0.65)	1.58 (-0.79)
6-month	1.48 (-1.02)	1.59 (-0.74)	1.58 (-0.79)
Sleep efficiency			
baseline	2.46	2.21	2.26
1-month	1.57 (-0.89)	1.66 (-0.55)	1.42 (-0.84)
3-month	1.36 (-1.10)	1.69 (-0.52)	1.27 (-0.99)
6-month	1.15 (-1.31)	1.55 (-0.66)	1.21 (-1.05)
Sleep disturbance			
baseline	1.41	1.33	1.32
1-month	0.98 (-0.43)	1.05 (-0.28)	0.92 (-0.40)
3-month	0.84 (-0.57)	1.05 (-0.28)	0.86 (-0.46)
6-month	0.91 (-0.50)	1.00 (-0.33)	0.83 (-0.49)
Daytime dysfunction			
baseline	2.27	2.25	2.26
1-month	1.38 (-0.89)	1.8 (-0.45)	1.71 (-0.55)
3-month	1.20 (-1.07)	1.76 (-0.49)	1.38 (-0.88)
6-month	1.24 (-1.03)	1.60 (-0.65)	1.20 (-1.06)
ESS score			
baseline	3.43	3.37	3.43
1-month	3.90 (+0.47)	3.97 (+0.60)	4.31 (+0.88)
3-month	3.49 (+0.06)	4.27 (+0.90)	3.83 (+0.40)
6-month	4.04 (+0.61)	4.06 (+0.69)	3.78 (+0.35)

© 2023 Lu M et al. *JAMA Network Open.*

GAD-7 score			
baseline	7.48	7.66	7.66
1-month	2.99 (-4.49)	5.16 (-2.50)	3.38 (-4.28)
3-month	2.02 (-5.46)	4.71 (-2.95)	2.45 (-5.21)
6-month	2.55 (-4.93)	4.16 (-3.50)	1.99 (-5.67)
PHQ-9 score			
baseline	8.79	8.61	8.72
1-month	3.46 (-5.33)	6.32 (-2.29)	4.24 (-4.48)
3-month	2.75 (-6.04)	5.93 (-2.68)	3.10 (-5.62)
6-month	3.3 (-5.49)	5.27 (-3.34)	2.64 (-6.08)
PHQ-15 score			
baseline	9.04	8.92	8.92
1-month	5.29 (-3.75)	7.33 (-1.59)	6.20 (-2.72)
3-month	4.62 (-4.42)	6.90 (-2.02)	5.17 (-3.75)
6-month	5.26 (-3.78)	6.65 (-2.27)	4.52 (-4.40)

Values in parentheses represent changes from baseline. A negative value indicates a decrease in the index, that is, an improvement in the patient's outcome. The green value indicates the improvement compared with baseline, and the red value indicates the deterioration.

eTable 4. Primary and Secondary Outcomes According to Treatment Group

Outcome	dCBT-I	Medication	Combination	p-value	SMD
PSQI score					
baseline	13.51 (3.03)	12.85 (3.49)	12.92 (3.49)	.02	0.135
1-month	8.31 (3.40)	9.27 (4.18)	8.21 (3.40)	<.001	0.186
3-month	7.30 (3.31)	9.30 (3.95)	7.29 (3.46)	<.001	0.366
6-month	7.15 (3.25)	8.92 (4.03)	6.98 (3.43)	<.001	0.351
Subjective sleep quality					
baseline	2.42 (0.71)	2.32 (0.80)	2.34 (0.80)	.20	0.085
1-month	1.18 (0.79)	1.44 (0.92)	1.19 (0.74)	<.001	0.211
3-month	1.01 (0.72)	1.44 (0.90)	1.06 (0.74)	<.001	0.36
6-month	1.15 (0.69)	1.47 (0.86)	1.04 (0.72)	<.001	0.368
Sleep onset latency					
baseline	2.47 (0.79)	2.40 (0.86)	2.37 (0.86)	.13	0.081
1-month	1.45 (0.88)	1.66 (0.97)	1.26 (0.85)	<.001	0.294
3-month	1.28 (0.83)	1.68 (0.89)	1.15 (0.82)	<.001	0.417
6-month	1.23 (0.79)	1.72 (0.96)	1.12 (0.80)	<.001	0.453
Sleep duration					
baseline	2.50 (0.94)	2.33 (1.05)	2.37 (1.01)	.05	0.11
1-month	1.77 (1.11)	1.65 (1.26)	1.71 (1.06)	.51	0.068
3-month	1.62 (1.06)	1.68 (1.20)	1.58 (1.04)	.30	0.06
6-month	1.48 (1.07)	1.59 (1.22)	1.58 (1.03)	.73	0.066
Sleep efficiency					
baseline	2.46 (0.88)	2.21 (1.08)	2.26 (1.07)	<.001	0.167
1-month	1.57 (1.10)	1.66 (1.16)	1.42 (1.08)	<.001	0.142
3-month	1.36 (1.05)	1.69 (1.17)	1.27 (1.06)	<.001	0.257
6-month	1.15 (1.12)	1.55 (1.18)	1.21 (1.04)	<.001	0.236

© 2023 Lu M et al. *JAMA Network Open.*

Sleep disturbance					
baseline	1.41 (0.54)	1.33 (0.60)	1.32 (0.58)	.16	0.098
1-month	0.98 (0.42)	1.05 (0.59)	0.92 (0.42)	<.001	0.181
3-month	0.84 (0.43)	1.05 (0.58)	0.86 (0.45)	<.001	0.27
6-month	0.91 (0.45)	1.00 (0.54)	0.83 (0.48)	<.001	0.219
Daytime dysfunction					
baseline	2.27 (0.92)	2.25 (0.91)	2.26 (0.93)	.97	0.008
1-month	1.38 (0.90)	1.80 (1.01)	1.71 (0.89)	<.001	0.307
3-month	1.20 (0.84)	1.76 (0.99)	1.38 (0.92)	<.001	0.403
6-month	1.24 (0.89)	1.60 (1.00)	1.20 (0.90)	<.001	0.285
ESS score					
baseline	3.43 (2.81)	3.37 (2.79)	3.43 (2.82)	.88	0.014
1-month	3.90 (3.54)	3.97 (3.89)	4.31 (3.66)	.13	0.074
3-month	3.49 (3.17)	4.27 (3.84)	3.83 (3.49)	.06	0.147
6-month	4.04 (3.81)	4.06 (3.81)	3.78 (3.40)	.56	0.051
GAD-7 score					
baseline	7.48 (5.88)	7.66 (5.86)	7.66 (5.82)	.93	0.021
1-month	2.99 (3.53)	5.16 (5.20)	3.38 (3.66)	<.001	0.331
3-month	2.02 (3.10)	4.71 (5.18)	2.45 (3.31)	<.001	0.429
6-month	2.55 (3.70)	4.16 (4.66)	1.99 (3.04)	<.001	0.368
PHQ-9 score					
baseline	8.79 (6.45)	8.61 (6.28)	8.72 (6.17)	.89	0.019
1-month	3.46 (3.61)	6.32 (6.02)	4.24 (4.09)	<.001	0.394
3-month	2.75 (2.99)	5.93 (6.04)	3.10 (3.69)	<.001	0.446
6-month	3.30 (4.57)	5.27 (5.69)	2.64 (3.39)	<.001	0.369
PHQ-15 score					
baseline	9.04 (4.90)	8.92 (4.52)	8.92 (4.59)	.96	0.018
1-month	5.29 (3.53)	7.33 (4.71)	6.20 (4.04)	<.001	0.329
3-month	4.62 (3.11)	6.90 (4.74)	5.17 (3.92)	<.001	0.374
6-month	5.26 (4.39)	6.65 (4.70)	4.52 (3.83)	<.001	0.327

eTable 5. Comparison of Primary and Secondary Outcomes in the dCBT-I and Medication Groups.

**Outcome**	**dCBT-I**	**Medication**	**p-value**	**SMD**	**Cohen's d (95% CI)**
PSQI score					
baseline	13.51 (3.03)	12.85 (3.49)	.006	0.203	0.21 (0.09 to 0.32)
1-month	8.31 (3.40)	9.27 (4.18)	.005	0.25	-0.26 (-0.38 to -0.14)
3-month	7.30 (3.31)	9.30 (3.95)	<.001	0.55	-0.57 (-0.68 to -0.45)
6-month	7.15 (3.25)	8.92 (4.03)	<.001	0.484	-0.5 (-0.62 to -0.38)
Subjective sleep quality					
baseline	2.42 (0.71)	2.32 (0.80)	.08	0.128	0.14 (0.02 to 0.25)
1-month	1.18 (0.79)	1.44 (0.92)	.001	0.313	-0.31 (-0.43 to -0.19)
3-month	1.01 (0.72)	1.44 (0.90)	<.001	0.539	-0.55 (-0.67 to -0.43)
6-month	1.15 (0.69)	1.47 (0.86)	<.001	0.415	-0.43 (-0.54 to -0.31)
Sleep onset latency					

© 2023 Lu M et al. *JAMA Network Open.*

baseline	2.47 (0.79)	2.40 (0.86)	.24	0.084	0.09 (-0.03 to 0.2)
1-month	1.45 (0.88)	1.66 (0.97)	.02	0.227	-0.23 (-0.35 to -0.11)
3-month	1.28 (0.83)	1.68 (0.89)	<.001	0.461	-0.47 (-0.59 to -0.35)
6-month	1.23 (0.79)	1.72 (0.96)	<.001	0.554	-0.58 (-0.7 to -0.46)
Sleep duration					
baseline	2.50 (0.94)	2.33 (1.05)	.02	0.164	0.17 (0.06 to 0.29)
1-month	1.77 (1.11)	1.65 (1.26)	.28	0.099	0.1 (-0.01 to 0.22)
3-month	1.62 (1.06)	1.68 (1.20)	.61	0.053	-0.05 (-0.17 to 0.06)
6-month	1.48 (1.07)	1.59 (1.22)	.48	0.095	-0.1 (-0.21 to 0.02)
Sleep efficiency					
baseline	2.46 (0.88)	2.21 (1.08)	<.001	0.251	0.26 (0.15 to 0.38)
1-month	1.57 (1.10)	1.66 (1.16)	.38	0.082	-0.08 (-0.2 to 0.04)
3-month	1.36 (1.05)	1.69 (1.17)	.005	0.305	-0.3 (-0.42 to -0.19)
6-month	1.15 (1.12)	1.55 (1.18)	.03	0.348	-0.35 (-0.47 to -0.23)
Sleep disturbance					
baseline	1.41 (0.54)	1.33 (0.60)	.13	0.126	0.14 (0.03 to 0.26)
1-month	0.98 (0.42)	1.05 (0.59)	.09	0.145	-0.15 (-0.26 to -0.03)
3-month	0.84 (0.43)	1.05 (0.58)	<.001	0.407	-0.43 (-0.55 to -0.32)
6-month	0.91 (0.45)	1.00 (0.54)	.17	0.184	-0.19 (-0.3 to -0.07)
Daytime dysfunction					
baseline	2.27 (0.92)	2.25 (0.91)	.88	0.012	0.02 (-0.09 to 0.14)
1-month	1.38 (0.90)	1.80 (1.01)	<.001	0.448	-0.45 (-0.57 to -0.33)
3-month	1.20 (0.84)	1.76 (0.99)	<.001	0.607	-0.63 (-0.75 to -0.51)
6-month	1.24 (0.89)	1.60 (1.00)	.005	0.38	-0.39 (-0.51 to -0.27)
ESS score					
baseline	3.43 (2.81)	3.37 (2.79)	.80	0.022	0.02 (-0.09 to 0.14)
1-month	3.90 (3.54)	3.97 (3.89)	.87	0.018	-0.02 (-0.14 to 0.1)
3-month	3.49 (3.17)	4.27 (3.84)	.04	0.221	-0.23 (-0.35 to -0.11)
6-month	4.04 (3.81)	4.06 (3.81)	.98	0.004	-0.01 (-0.12 to 0.11)
GAD-7 score					
baseline	7.48 (5.88)	7.66 (5.86)	.72	0.031	-0.03 (-0.15 to 0.09)
1-month	2.99 (3.53)	5.16 (5.20)	<.001	0.489	-0.52 (-0.64 to -0.4)
3-month	2.02 (3.10)	4.71 (5.18)	<.001	0.632	-0.69 (-0.81 to -0.57)
6-month	2.55 (3.70)	4.16 (4.66)	.002	0.384	-0.4 (-0.52 to -0.28)
PHQ-9 score					
baseline	8.79 (6.45)	8.61 (6.28)	.75	0.028	0.03 (-0.09 to 0.14)
1-month	3.46 (3.61)	6.32 (6.02)	<.001	0.576	-0.63 (-0.75 to -0.51)
3-month	2.75 (2.99)	5.93 (6.04)	<.001	0.667	-0.75 (-0.87 to -0.63)
6-month	3.30 (4.57)	5.27 (5.69)	.004	0.381	-0.4 (-0.51 to -0.28)
PHQ-15 score					
baseline	9.04 (4.90)	8.92 (4.52)	.78	0.027	0.03 (-0.09 to 0.14)
1-month	5.29 (3.53)	7.33 (4.71)	<.001	0.49	-0.52 (-0.63 to -0.4)
3-month	4.62 (3.11)	6.90 (4.74)	<.001	0.57	-0.61 (-0.73 to -0.49)
6-month	5.26 (4.39)	6.65 (4.70)	.03	0.304	-0.31 (-0.43 to -0.19)

© 2023 Lu M et al. *JAMA Network Open.*

eTable 6. Comparison of Primary and Secondary Outcomes in the Medication and Combination Groups.

Outcome	Medication	Combination	p-value	SMD	Cohen's d (95% CI)
PSQI score					
baseline	12.85 (3.49)	12.92 (3.49)	.65	0.019	-0.02 (-0.1 to 0.06)
1-month	9.27 (4.18)	8.21 (3.40)	<.001	0.278	0.26 (0.19 to 0.34)
3-month	9.30 (3.95)	7.29 (3.46)	<.001	0.544	0.52 (0.45 to 0.6)
6-month	8.92 (4.03)	6.98 (3.43)	<.001	0.518	0.5 (0.42 to 0.58)
Subjective sleep quality					
baseline	2.32 (0.80)	2.34 (0.80)	.68	0.018	-0.03 (-0.1 to 0.05)
1-month	1.44 (0.92)	1.19 (0.74)	<.001	0.307	0.28 (0.21 to 0.36)
3-month	1.44 (0.90)	1.06 (0.74)	<.001	0.47	0.44 (0.36 to 0.52)
6-month	1.47 (0.86)	1.04 (0.72)	<.001	0.541	0.52 (0.44 to 0.6)
Sleep onset latency					
baseline	2.40 (0.86)	2.37 (0.86)	.40	0.037	0.03 (-0.04 to 0.11)
1-month	1.66 (0.97)	1.26 (0.85)	<.001	0.437	0.42 (0.35 to 0.5)
3-month	1.68 (0.89)	1.15 (0.82)	<.001	0.624	0.61 (0.53 to 0.68)
6-month	1.72 (0.96)	1.12 (0.80)	<.001	0.671	0.65 (0.57 to 0.73)
Sleep duration					
baseline	2.33 (1.05)	2.37 (1.01)	.38	0.037	-0.04 (-0.11 to 0.04)
1-month	1.65 (1.26)	1.71 (1.06)	.35	0.053	-0.05 (-0.13 to 0.03)
3-month	1.68 (1.20)	1.58 (1.04)	.13	0.089	0.09 (0.01 to 0.16)
6-month	1.59 (1.22)	1.58 (1.03)	.93	0.007	0.01 (-0.07 to 0.08)
Sleep efficiency					
baseline	2.21 (1.08)	2.26 (1.07)	.31	0.043	-0.05 (-0.12 to 0.03)
1-month	1.66 (1.16)	1.42 (1.08)	<.001	0.211	0.21 (0.13 to 0.29)
3-month	1.69 (1.17)	1.27 (1.06)	<.001	0.384	0.37 (0.29 to 0.44)
6-month	1.55 (1.18)	1.21 (1.04)	<.001	0.306	0.3 (0.22 to 0.37)
Sleep disturbance					
baseline	1.33 (0.60)	1.32 (0.58)	.65	0.019	0.02 (-0.06 to 0.09)
1-month	1.05 (0.59)	0.92 (0.42)	<.001	0.26	0.23 (0.16 to 0.31)
3-month	1.05 (0.58)	0.86 (0.45)	<.001	0.373	0.34 (0.27 to 0.42)
6-month	1.00 (0.54)	0.83 (0.48)	<.001	0.32	0.32 (0.25 to 0.4)
Daytime dysfunction					
baseline	2.25 (0.91)	2.26 (0.93)	.81	0.01	-0.01 (-0.09 to 0.07)
1-month	1.80 (1.01)	1.71 (0.89)	.07	0.101	0.09 (0.02 to 0.17)
3-month	1.76 (0.99)	1.38 (0.92)	<.001	0.395	0.39 (0.31 to 0.47)
6-month	1.60 (1.00)	1.20 (0.90)	<.001	0.425	0.41 (0.33 to 0.49)
ESS score					
baseline	3.37 (2.79)	3.43 (2.82)	.62	0.02	-0.02 (-0.1 to 0.05)
1-month	3.97 (3.89)	4.31 (3.66)	.08	0.091	-0.09 (-0.16 to -0.01)
3-month	4.27 (3.84)	3.83 (3.49)	.04	0.119	0.12 (0.04 to 0.19)
6-month	4.06 (3.81)	3.78 (3.40)	.33	0.077	0.08 (0 to 0.15)
GAD-7 score					
baseline	7.66 (5.86)	7.66 (5.82)	.97	0.001	0 (-0.08 to 0.08)

© 2023 Lu M et al. *JAMA Network Open.*

1-month	5.16 (5.20)	3.38 (3.66)	<.001	0.397	0.37 (0.29 to 0.44)
3-month	4.71 (5.18)	2.45 (3.31)	<.001	0.52	0.47 (0.39 to 0.55)
6-month	4.16 (4.66)	1.99 (3.04)	<.001	0.553	0.5 (0.42 to 0.58)
PHQ-9 score					
baseline	8.61 (6.28)	8.72 (6.17)	.66	0.017	-0.02 (-0.09 to 0.06)
1-month	6.32 (6.02)	4.24 (4.09)	<.001	0.403	0.37 (0.29 to 0.45)
3-month	5.93 (6.04)	3.10 (3.69)	<.001	0.567	0.51 (0.43 to 0.59)
6-month	5.27 (5.69)	2.64 (3.39)	<.001	0.561	0.5 (0.43 to 0.58)
PHQ-15 score					
baseline	8.92 (4.52)	8.92 (4.59)	0.98	0.001	0 (-0.08 to 0.08)
1-month	7.33 (4.71)	6.20 (4.04)	<.001	0.257	0.25 (0.17 to 0.32)
3-month	6.90 (4.74)	5.17 (3.92)	<.001	0.398	0.38 (0.3 to 0.46)
6-month	6.65 (4.70)	4.52 (3.83)	<.001	0.496	0.47 (0.4 to 0.55)

eTable 7. Comparison of Primary and Secondary Outcomes in the dCBT-I and Combination Groups.

Outcome	dCBT-I	Combination	p-value	SMD	Cohen's d (95% CI)
PSQI score					
baseline	13.51 (3.03)	12.92 (3.49)	.006	0.182	0.19 (0.09 to 0.29)
1-month	8.31 (3.40)	8.21 (3.40)	.70	0.031	0.03 (-0.07 to 0.13)
3-month	7.30 (3.31)	7.29 (3.46)	.96	0.005	0 (-0.1 to 0.11)
6-month	7.15 (3.25)	6.98 (3.43)	.66	0.05	0.05 (-0.05 to 0.15)
Subjective sleep quality					
baseline	2.42 (0.71)	2.34 (0.80)	.10	0.109	0.11 (0.01 to 0.21)
1-month	1.18 (0.79)	1.19 (0.74)	.87	0.015	-0.01 (-0.12 to 0.09)
3-month	1.01 (0.72)	1.06 (0.74)	.41	0.073	-0.07 (-0.17 to 0.03)
6-month	1.15 (0.69)	1.04 (0.72)	.15	0.149	0.16 (0.06 to 0.26)
Sleep onset latency					
baseline	2.47 (0.79)	2.37 (0.86)	.05	0.122	0.13 (0.02 to 0.23)
1-month	1.45 (0.88)	1.26 (0.85)	.02	0.219	0.22 (0.11 to 0.32)
3-month	1.28 (0.83)	1.15 (0.82)	.08	0.167	0.16 (0.05 to 0.26)
6-month	1.23 (0.79)	1.12 (0.80)	.19	0.134	0.14 (0.04 to 0.24)
Sleep duration					
baseline	2.50 (0.94)	2.37 (1.01)	.04	0.128	0.14 (0.03 to 0.24)
1-month	1.77 (1.11)	1.71 (1.06)	.54	0.052	0.05 (-0.05 to 0.16)
3-month	1.62 (1.06)	1.58 (1.04)	.70	0.038	0.04 (-0.06 to 0.14)
6-month	1.48 (1.07)	1.58 (1.03)	.44	0.096	-0.09 (-0.2 to 0.01)
Sleep efficiency					
baseline	2.46 (0.88)	2.26 (1.07)	<.001	0.206	0.22 (0.12 to 0.32)
1-month	1.57 (1.10)	1.42 (1.08)	.12	0.132	0.14 (0.03 to 0.24)
3-month	1.36 (1.05)	1.27 (1.06)	.41	0.083	0.09 (-0.02 to 0.19)
6-month	1.15 (1.12)	1.21 (1.04)	.71	0.055	-0.05 (-0.16 to 0.05)
Sleep disturbance					
baseline	1.41 (0.54)	1.32 (0.58)	.06	0.147	0.16 (0.06 to 0.27)
1-month	0.98 (0.42)	0.92 (0.42)	.10	0.139	0.14 (0.04 to 0.25)

© 2023 Lu M et al. *JAMA Network Open.*

3-month	0.84 (0.43)	0.86 (0.45)	.77	0.031	-0.05 (-0.15 to 0.06)
6-month	0.91 (0.45)	0.83 (0.48)	.20	0.155	0.18 (0.07 to 0.28)
Daytime dysfunction					
baseline	2.27 (0.92)	2.26 (0.93)	.98	0.002	0.01 (-0.09 to 0.11)
1-month	1.38 (0.90)	1.71 (0.89)	<.001	0.37	-0.37 (-0.47 to -0.26)
3-month	1.20 (0.84)	1.38 (0.92)	.04	0.206	-0.21 (-0.31 to -0.11)
6-month	1.24 (0.89)	1.20 (0.90)	.68	0.05	0.04 (-0.06 to 0.15)
ESS score					
baseline	3.43 (2.81)	3.43 (2.82)	.99	0.001	0 (-0.1 to 0.1)
1-month	3.90 (3.54)	4.31 (3.66)	.26	0.114	-0.12 (-0.22 to -0.01)
3-month	3.49 (3.17)	3.83 (3.49)	.29	0.102	-0.11 (-0.21 to 0)
6-month	4.04 (3.81)	3.78 (3.40)	.63	0.073	0.07 (-0.03 to 0.17)
GAD-7 score					
baseline	7.48 (5.88)	7.66 (5.82)	.72	0.03	-0.03 (-0.13 to 0.07)
1-month	2.99 (3.53)	3.38 (3.66)	.17	0.109	-0.11 (-0.21 to -0.01)
3-month	2.02 (3.10)	2.45 (3.31)	.21	0.136	-0.14 (-0.24 to -0.03)
6-month	2.55 (3.70)	1.99 (3.04)	.18	0.166	0.15 (0.05 to 0.26)
PHQ-9 score					
baseline	8.79 (6.45)	8.72 (6.17)	.89	0.011	0.01 (-0.09 to 0.11)
1-month	3.46 (3.61)	4.24 (4.09)	.007	0.203	-0.21 (-0.31 to -0.11)
3-month	2.75 (2.99)	3.10 (3.69)	.24	0.103	-0.11 (-0.22 to -0.01)
6-month	3.30 (4.57)	2.64 (3.39)	.25	0.165	0.15 (0.05 to 0.25)
PHQ-15 score					
baseline	9.04 (4.90)	8.92 (4.59)	.77	0.026	0.02 (-0.08 to 0.13)
1-month	5.29 (3.53)	6.20 (4.04)	.001	0.241	-0.25 (-0.36 to -0.15)
3-month	4.62 (3.11)	5.17 (3.92)	.07	0.156	-0.17 (-0.27 to -0.07)
6-month	5.26 (4.39)	4.52 (3.83)	.18	0.182	0.17 (0.07 to 0.27)

eTable 8. Time Series of Primary and Secondary Outcomes.

**Outcome**	**time (month)**	**dCBT-I**	**Medication**	**Combination**
PSQI score	0	13.51	12.85	12.92
	1	8.31 (-5.2)	9.27 (-3.58)	8.21 (-4.71)
	2	7.89 (-0.42)	9.36 (+0.09)	7.75 (-0.46)
	3	7.3 (-0.59)	9.3 (-0.06)	7.29 (-0.46)
	4	7.2 (-0.1)	8.92 (-0.38)	7.08 (-0.21)
	5	6.91 (-0.29)	8.63 (-0.29)	7.07 (-0.01)
	6	7.15 (+0.24)	8.92 (+0.29)	6.98 (-0.09)
Subjective sleep quality	0	2.42	2.32	2.34
	1	1.18 (-1.24)	1.44 (-0.88)	1.19 (-1.15)
	2	1.1 (-0.08)	1.44 (0)	1.12 (-0.07)
	3	1.01 (-0.09)	1.44 (0)	1.06 (-0.06)
	4	1.1 (+0.09)	1.43 (-0.01)	1.04 (-0.02)
	5	1.18 (+0.08)	1.4 (-0.03)	1.04 (0)
	6	1.15 (-0.03)	1.47 (+0.07)	1.04 (0)

© 2023 Lu M et al. *JAMA Network Open.*

Sleep onset latency	0	2.47	2.4	2.37
	1	1.45 (-1.02)	1.66 (-0.74)	1.26 (-1.11)
	2	1.38 (-0.07)	1.7 (+0.04)	1.21 (-0.05)
	3	1.28 (-0.1)	1.68 (-0.02)	1.15 (-0.06)
	4	1.3 (+0.02)	1.62 (-0.06)	1.12 (-0.03)
	5	1.23 (-0.07)	1.63 (+0.01)	1.14 (+0.02)
	6	1.23 (0)	1.72 (+0.09)	1.12 (-0.02)
Sleep duration	0	2.5	2.33	2.37
	1	1.77 (-0.73)	1.65 (-0.68)	1.71 (-0.66)
	2	1.7 (-0.07)	1.66 (+0.01)	1.64 (-0.07)
	3	1.62 (-0.08)	1.68 (+0.02)	1.58 (-0.06)
	4	1.6 (-0.02)	1.57 (-0.11)	1.56 (-0.02)
	5	1.47 (-0.13)	1.5 (-0.07)	1.57 (+0.01)
	6	1.48 (+0.01)	1.59 (+0.09)	1.58 (+0.01)
Sleep efficiency	0	2.46	2.21	2.26
	1	1.57 (-0.89)	1.66 (-0.55)	1.42 (-0.84)
	2	1.46 (-0.11)	1.7 (+0.04)	1.35 (-0.07)
	3	1.36 (-0.1)	1.69 (-0.01)	1.27 (-0.08)
	4	1.26 (-0.1)	1.54 (-0.15)	1.24 (-0.03)
	5	1.09 (-0.17)	1.47 (-0.07)	1.23 (-0.01)
	6	1.15 (+0.06)	1.55 (+0.08)	1.21 (-0.02)
Sleep disturbance	0	1.41	1.33	1.32
	1	0.98 (-0.43)	1.05 (-0.28)	0.92 (-0.4)
	2	0.92 (-0.06)	1.07 (+0.02)	0.89 (-0.03)
	3	0.84 (-0.08)	1.05 (-0.02)	0.86 (-0.03)
	4	0.78 (-0.06)	1.04 (-0.01)	0.83 (-0.03)
	5	0.76 (-0.02)	1.02 (-0.02)	0.84 (+0.01)
	6	0.91 (+0.15)	1 (-0.02)	0.83 (-0.01)
Sleep medication use	0	1.51	2.11	2.08
	1	0.14 (-1.37)	2.65 (+0.54)	2.51 (+0.43)
	2	0.12 (-0.02)	2.68 (+0.03)	2.45 (-0.06)
	3	0.13 (+0.01)	2.6 (-0.08)	2.33 (-0.12)
	4	0.09 (-0.04)	2.52 (-0.08)	2.22 (-0.11)
	5	0.07 (-0.02)	2.33 (-0.19)	2.08 (-0.14)
	6	0.06 (-0.01)	2.11 (-0.22)	1.93 (-0.15)
Daytime dysfunction	0	2.27	2.25	2.26
	1	1.38 (-0.89)	1.8 (-0.45)	1.71 (-0.55)
	2	1.33 (-0.05)	1.78 (-0.02)	1.54 (-0.17)
	3	1.2 (-0.13)	1.76 (-0.02)	1.38 (-0.16)
	4	1.15 (-0.05)	1.72 (-0.04)	1.29 (-0.09)
	5	1.18 (+0.03)	1.6 (-0.12)	1.25 (-0.04)
	6	1.24 (+0.06)	1.6 (0)	1.2 (-0.05)
ESS score	0	3.43	3.37	3.43
	1	3.9 (+0.47)	3.97 (+0.6)	4.31 (+0.88)
	2	3.77 (-0.13)	4.1 (+0.13)	4.04 (-0.27)

© 2023 Lu M et al. *JAMA Network Open.*

	3	3.49 (-0.28)	4.27 (+0.17)	3.83 (-0.21)
	4	3.43 (-0.06)	4.03 (-0.24)	3.63 (-0.2)
	5	3.6 (+0.17)	3.97 (-0.06)	3.76 (+0.13)
	6	4.04 (+0.44)	4.06 (+0.09)	3.78 (+0.02)
GAD-7 score	0	7.48	7.66	7.66
	1	2.99 (-4.49)	5.16 (-2.5)	3.38 (-4.28)
	2	2.63 (-0.36)	5.07 (-0.09)	2.92 (-0.46)
	3	2.02 (-0.61)	4.71 (-0.36)	2.45 (-0.47)
	4	2.09 (+0.07)	4.28 (-0.43)	2.19 (-0.26)
	5	2.44 (+0.35)	3.89 (-0.39)	2.15 (-0.04)
	6	2.55 (+0.11)	4.16 (+0.27)	1.99 (-0.16)
PHQ-9 score	0	8.79	8.61	8.72
	1	3.46 (-5.33)	6.32 (-2.29)	4.24 (-4.48)
	2	3.1 (-0.36)	6.28 (-0.04)	3.68 (-0.56)
	3	2.75 (-0.35)	5.93 (-0.35)	3.1 (-0.58)
	4	2.67 (-0.08)	5.56 (-0.37)	2.84 (-0.26)
	5	2.85 (+0.18)	5.06 (-0.5)	2.82 (-0.02)
	6	3.3 (+0.45)	5.27 (+0.21)	2.64 (-0.18)
PHQ-15 score	0	9.04	8.92	8.92
	1	5.29 (-3.75)	7.33 (-1.59)	6.2 (-2.72)
	2	5.04 (-0.25)	7.29 (-0.04)	5.72 (-0.48)
	3	4.62 (-0.42)	6.9 (-0.39)	5.17 (-0.55)
	4	4.4 (-0.22)	6.63 (-0.27)	4.79 (-0.38)
	5	4.6 (+0.2)	6.61 (-0.02)	4.7 (-0.09)
	6	5.26 (+0.66)	6.65 (+0.04)	4.52 (-0.18)

Outcomes in each month were calculated from visible recorded data. Values in parentheses represent changes from the previous time point. A negative value indicates a decrease in the index, that is, an improvement in the patient's outcome.

eTable 9. Patient Engagement in Each dCBT-I Session.

Month	SH	RT	SR	SC	CR
1	56.02%	94.14%	80.03%	8.78%	8.93%
2	29.66%	82.38%	77.34%	27.90%	6.96%
3	17.21%	72.16%	66.71%	32.79%	23.89%
4	12.66%	64.39%	58.21%	15.14%	28.62%
5	9.66%	57.59%	49.03%	9.94%	17.93%
6	8.56%	49.91%	42.66%	10.72%	12.79%
Overall	68.71%	98.40%	92.07%	48.68%	44.33%

Each value indicates the proportion of patients who completed the session in that month. The overall value indicates the percentage of patient engagement in the whole 6-month follow-up for each dCBT-I session.

eFigure 1. **Study Design**.

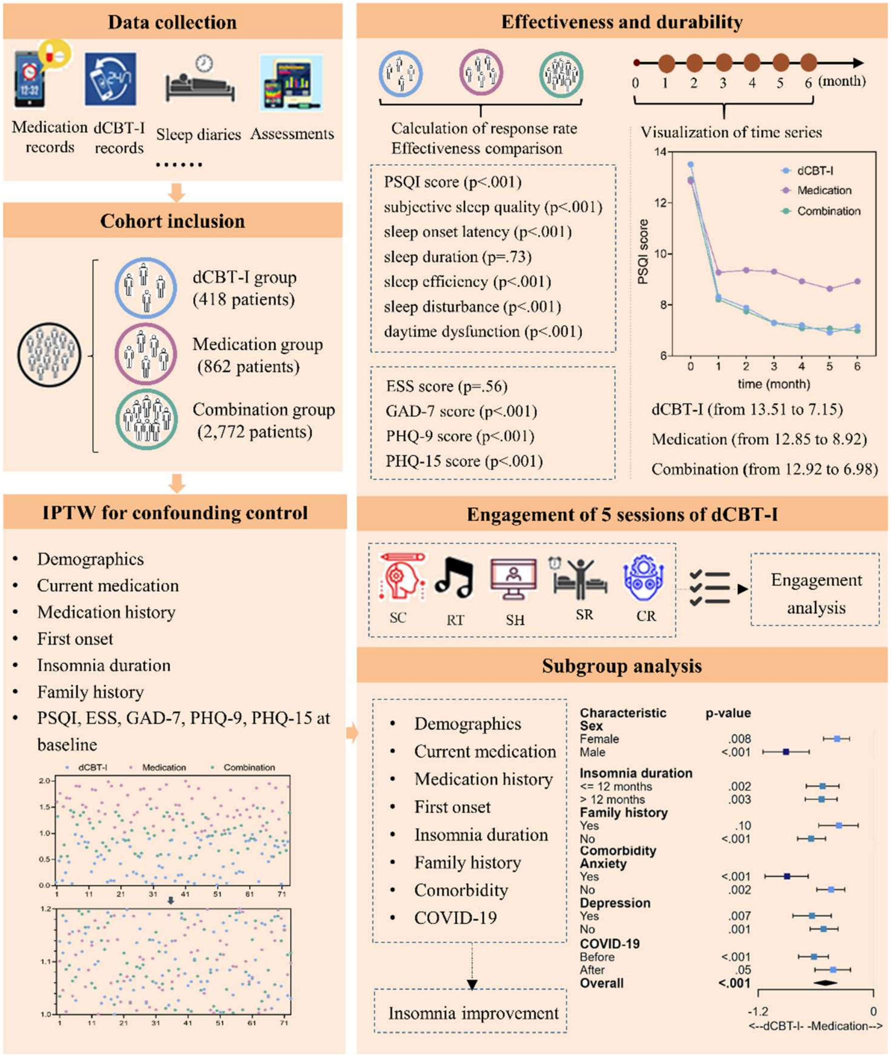

(1) Real-world data (including medication records, dCBT-I records, sleep diaries, assessments, etc.) were collected by a mobile App called Good Sleep 365. (2) Patients were assigned to three cohorts based on prescriptions, namely dCBT-I, drug therapy, and their combination. (3) IPTW was applied to control confounders that influence intervention choices and eliminate between-group differences. After IPTW adjustment, the covariate distributions of the three groups were balanced. (4) The primary and secondary outcomes were compared to assess the effectiveness of the three treatment modalities, with p<.05 indicating a significant difference in the target outcome between the three groups. Response rates were calculated to estimate their effectiveness. The durability of the three treatment modalities on the results was then evaluated. (5) The proportion of patients who completed different dCBT-I sessions was used to analyze patient engagement. (6) Cohen's d effect size and p-value were also calculated to analyze the effectiveness of three modes in sub-populations with different characteristics.

Abbreviations: dCBT-I, digital cognitive behavioral therapy for Insomnia; PSQI, Pittsburgh sleep quality index; ESS, Epworth sleepiness score; GAD-7, generalized anxiety disorder 7; PHQ-9, patient health questionnaire 9; PHQ-15, patient health questionnaire 15; SC, stimulus control; RT, relaxation training; SH, sleep hygiene; SR, sleep restriction; CR, cognitive reconstruction.

eFigure 2. **Interfaces of Good Sleep 365 App. (A) Home Page. (B) CBT-I Training Page. (C) Sleep Diary Page.**

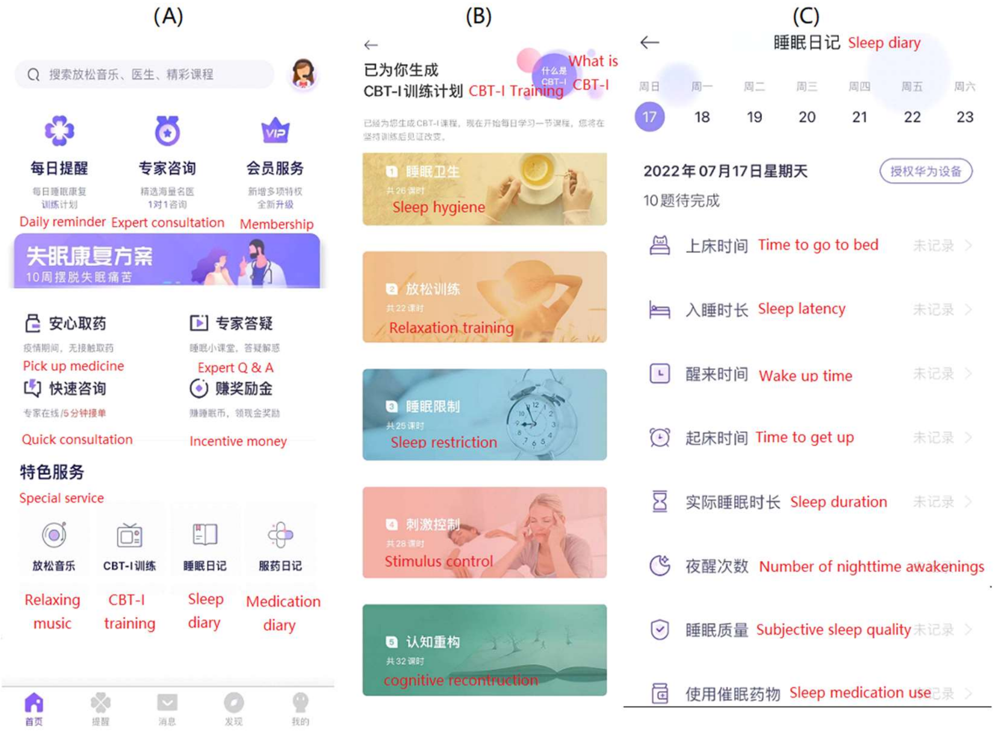

Good Sleep 365 is a mobile application that provides insomnia-assisted diagnosis and treatment services, aiming to create an efficient and safe online environment. It provides interfaces for CBT-I training, medication recording, sleep assessment, doctor consultation, and more.

When patients seek medical treatment for the first time, they need to register for the Good Sleep 365 App, pay for course access, and fill in their personal information. After logging in, the unified homepage is presented (eFigure 2 (A)), and each button corresponds to a different function. The patient clicks the relaxing music and CBT-I training buttons to complete the relevant courses of CBT-I according to the doctor's instructions (eFigure 2 (B)). Courses are systematically tailored by physicians following clinical guidelines, which are grouped into dCBT-I sessions as a 3-month program. An overview video is first provided to introduce the preliminary knowledge of dCBT-I. For each dCBT-I session, instructions are provided to help recipients understand it smoothly. The content of each course is presented in the form of video or audio and is approximately 30-50 minutes long. Despite it was encouraged to complete all dCBT-I sessions one after another in sequence, recipients have the flexibility to choose some sessions they were interested in or considered more helpful. Recipients could also revisit the courses in the App if they preferred to.

Psychiatrists will add the medication list to the app, including the name of the medication, the dosage, and the instruction on how to take it. The patient clicks the medication diary button to record the daily hypnotic medication. Regular sleep diaries (eFigure 2 (C)) and assessments are used to track and customize behavioral therapy, such as an automatic alarm clock function that reminds patients of bedtime and wake-up times.

© 2023 Lu M et al. *JAMA Network Open.*

eFigure 3. Forest Plots of Primary and Secondary Outcomes for Medication Therapy vs Combination Therapy.

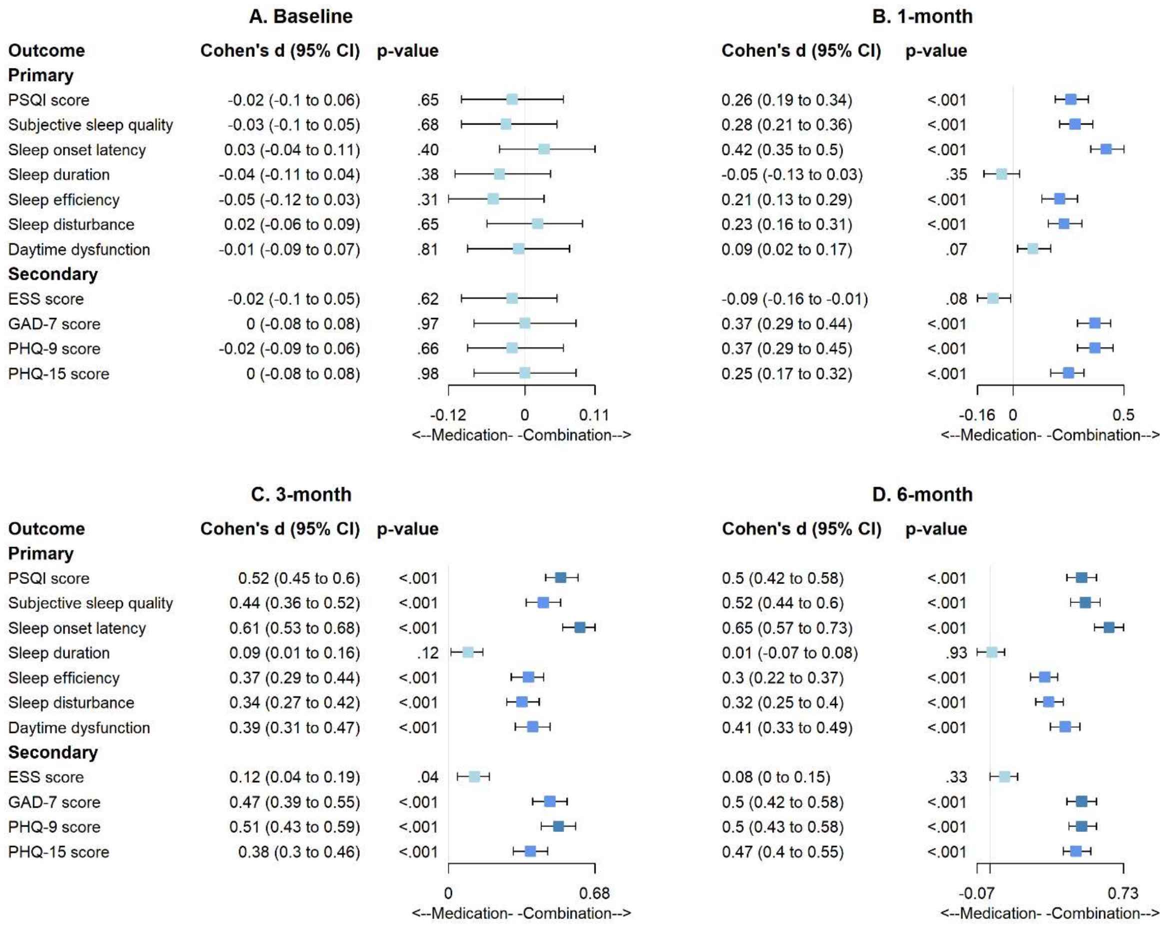

Each horizontal line on a forest plot represents the 95% confidence interval of outcome comparison (Cohen's d effect size), and the result is plotted as a box in the middle of the horizontal line. The vertical line is a reference line, placed at the value where there is no difference between the two interventions. The position of the box relative to the vertical line reflects the favorite (more effective) intervention mode. The box to the left of the vertical line indicates that the left treatment mode is superior to the right treatment mode shown at the bottom of the plot. Small, moderate, and large effect sizes are displayed in different colors, with darker colors indicating larger effect sizes.

eFigure 4. Forest Plots of Primary and Secondary Outcomes for dCBT-I vs Combination Therapy.

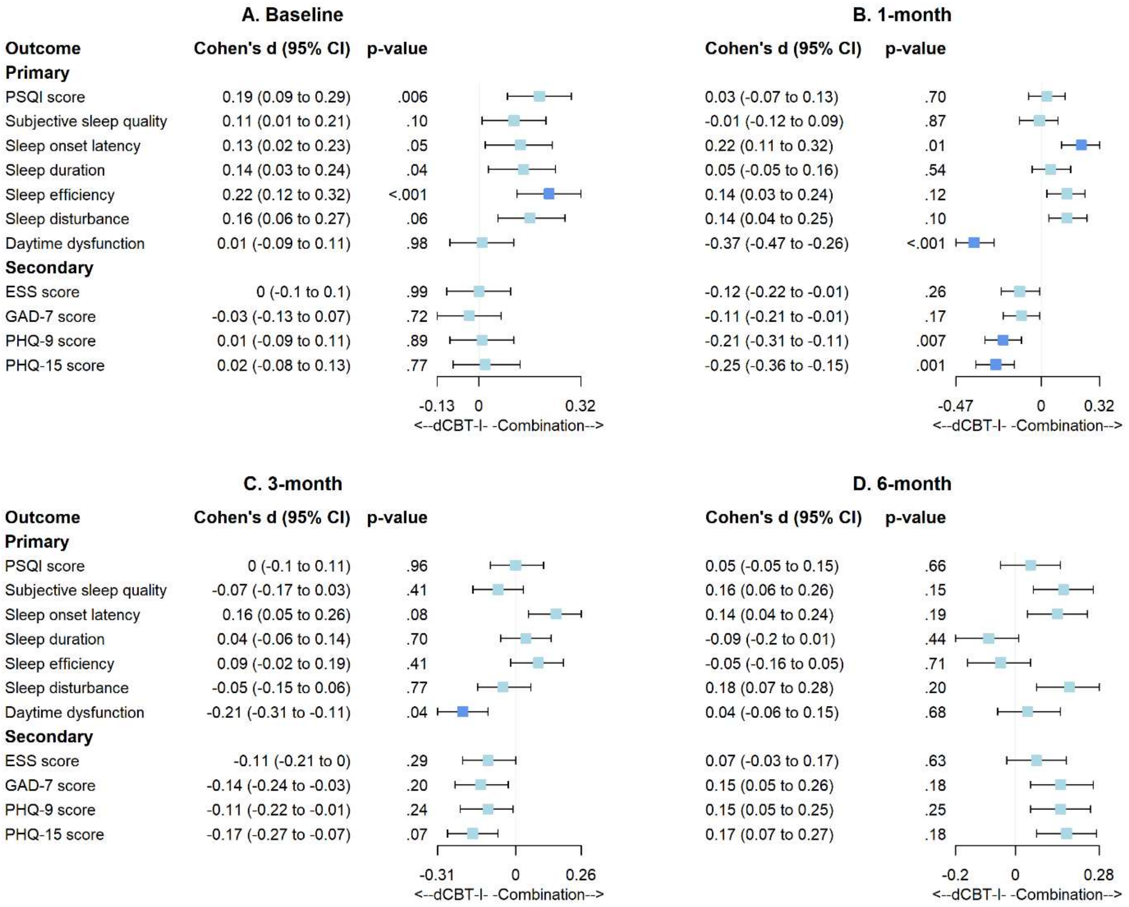

Each horizontal line on a forest plot represents the 95% confidence interval of outcome comparison (Cohen's d effect size), and the result is plotted as a box in the middle of the horizontal line. The vertical line is a reference line, placed at the value where there is no difference between the two interventions. The position of the box relative to the vertical line reflects the favorite (more effective) intervention mode. The box to the left of the vertical line indicates that the left treatment mode is superior to the right treatment mode shown at the bottom of the plot. Small, moderate, and large effect sizes are displayed in different colors, with darker colors indicating larger effect sizes.

© 2023 Lu M et al. *JAMA Network Open.*

eFigure 5. Line Charts of Time Series of the Primary and Secondary Outcomes During a 6-Month Follow-up.

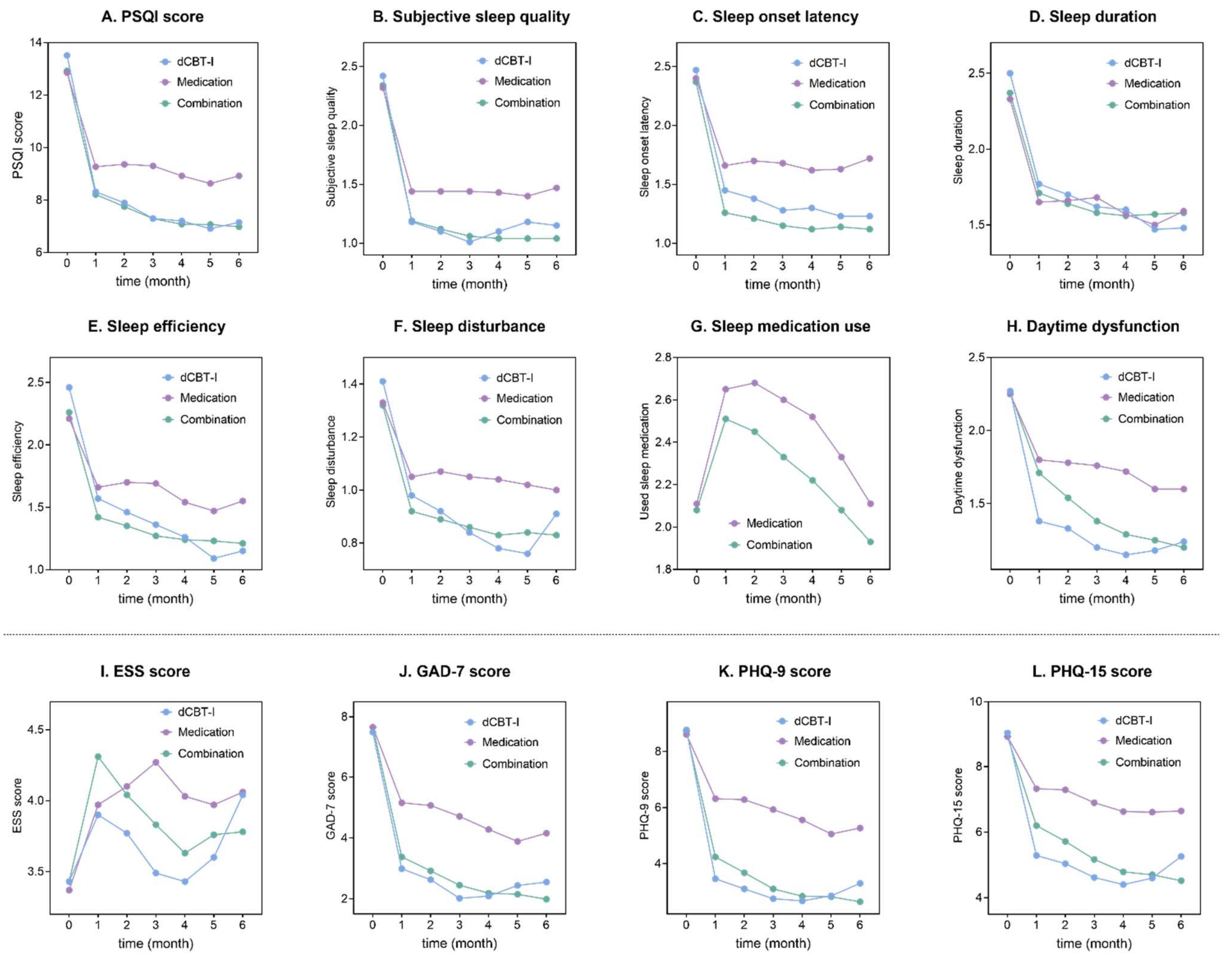

The time series consisted of outcome values for different groups at 0, 1, 2, 3, 4, 5, and 6 months after treatment. PSQI includes 7 subitems, namely subjective sleep quality (PSQI part A), sleep onset latency (PSQI part B, i.e., how long it takes to fall asleep), sleep duration (PSQI part C), sleep efficiency (PSQI part D, i.e., the percentage of time a person spends in bed), sleep disturbance (PSQI part E), sleep medication use (PSQI part F), and daytime dysfunction (PSQI part G), and each sub-item ranges from 0 to 3. The subplots B-H correspond to the time series of PSQI subitems. A decrease in the score symbolizes an improvement in the corresponding outcome.

eFigure 6. Patient Engagement in Each of the 5 dCBT-I Sessions: (a) Monthly Engagement; (b) Overall Engagement up to 6-month Follow-up.

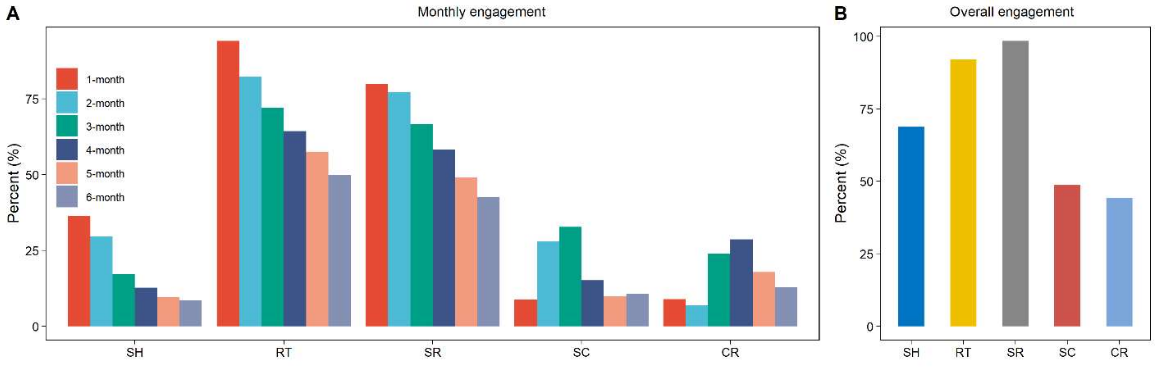

The monthly engagement illustrates the fluctuation of completion rate of each session by month. The overall engagement shows the overall completion rate of each session over a 6-month period.

© 2023 Lu M et al. *JAMA Network Open.*

eFigure 7. Subgroup Analysis Comparing the Effectiveness of Medication Therapy and Combination Therapy.

Characteristic	Cohen's d (95%CI)	p-value
**Sex**		
Female	0.43 (0.34 to 0.52)	<.001
Male	0.82 (0.67 to 0.98)	<.001
**Age**		
18-55	0.57 (0.48 to 0.65)	<.001
56-80	0.31 (0.13 to 0.48)	.07
**Education**		
Low	0.33 (0.23 to 0.44)	<.001
High	0.79 (0.67 to 0.91)	<.001
**Employment**		
Employed	0.58 (0.50 to 0.67)	<.001
Unemployed	0.23 (0.04 to 0.42)	.20
**Insomnia duration**		
<= 12 months	0.56 (0.44 to 0.67)	<.001
> 12 months	0.55 (0.44 to 0.65)	<.001
**Family history**		
Yes	0.32 (0.18 to 0.46)	.01
No	0.65 (0.55 to 0.74)	<.001
**First onset**		
Yes	0.53 (0.38 to 0.69)	<.001
No	0.53 (0.44 to 0.62)	<.001
**Medication history**		
Yes	0.63 (0.53 to 0.73)	<.001
No	0.41 (0.29 to 0.54)	<.001
**Current medication**		
Yes	0.54 (0.45 to 0.62)	<.001
No	0.56 (0.40 to 0.72)	<.001
**Comorbidity**		
**Anxiety**		
Yes	0.61 (0.49 to 0.74)	<.001
No	0.49 (0.39 to 0.59)	<.001
**Depression**		
Yes	0.50 (0.38 to 0.62)	<.001
No	0.53 (0.43 to 0.64)	<.001
**Somatic symptoms**		
Yes	0.44 (0.33 to 0.55)	<.001
No	0.60 (0.49 to 0.70)	<.001
**COVID-19**		
Before	0.57 (0.46 to 0.68)	<.001
After	0.53 (0.42 to 0.64)	<.001
**Overall**	**0.5 (0.42 to 0.58)**	**<.001**

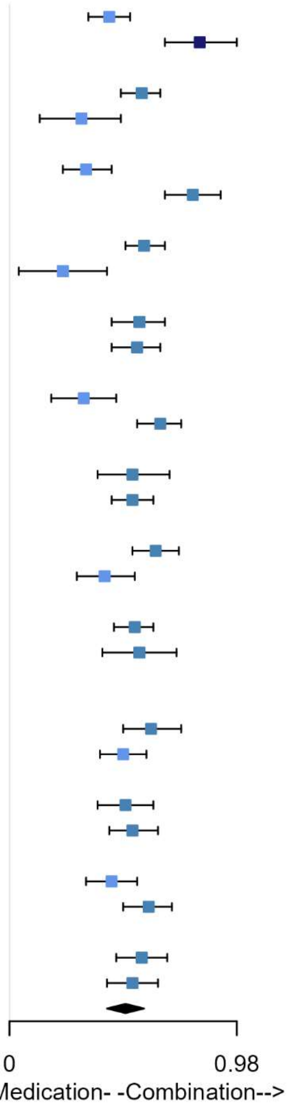

Each horizontal line on a forest plot represents the 95% confidence interval of comparison (Cohen's d effect size) of PSQI score at month 6 in a subgroup, and the result is plotted as a box in the middle of the horizontal line. The last row indicates the comparison of effectiveness on the whole population. The vertical line is a reference line, placed at the value where there is no difference between two interventions. The position of the box relative to the vertical line reflects the favorite (more effective) intervention mode. The box to the left of the vertical line indicates that the left treatment mode is superior to the right treatment mode shown at the bottom of the plot. Small, moderate, and large effect sizes are displayed in different colors, with darker colors indicating larger effect sizes. Education level and insomnia duration were coarsely categorized to avoid over-classification resulting in a smaller sample to influence confounders' control with IPTW. Patients with primary, middle and senior education were classified as low educated, while patients with higher education were classified as high educated.

Insomnia duration: Length of interval from the first occurrence of insomnia to baseline. First onset: Whether this insomnia was the first attack. Medication history: Whether the patient had a history of taking any form of hypnotic medications prior to baseline. Current medication: Whether the patient was still taking any form of hypnotic medications currently at baseline.

© 2023 Lu M et al. *JAMA Network Open.*

eFigure 8. Subgroup Analysis Comparing the Effectiveness of dCBT-I and Combination Therapy.

Characteristic	Cohen's d (95%CI)	p-value
**Sex**		
Female	0.10 (-0.02 to 0.22)	.44
Male	-0.03 (-0.22 to 0.17)	.91
**Age**		
18-55	0.13 (0.02 to 0.24)	.32
56-80	-0.40 (-0.68 to -0.11)	.07
**Education**		
Low	-0.08 (-0.24 to 0.09)	.65
High	0.18 (0.04 to 0.31)	.20
**Employment**		
Employed	0.14 (0.04 to 0.25)	.21
Unemployed	-0.58 (-0.91 to -0.26)	.07
**Insomnia duration**		
<= 12 months	0.10 (-0.04 to 0.25)	.50
> 12 months	0.07 (-0.07 to 0.21)	.67
**Family history**		
Yes	-0.01 (-0.18 to 0.16)	.96
No	0.08 (-0.05 to 0.21)	.54
**First onset**		
Yes	0.19 (-0.01 to 0.39)	.32
No	0.04 (-0.08 to 0.16)	.78
**Medication history**		
Yes	-0.05 (-0.20 to 0.11)	.74
No	0.26 (0.12 to 0.40)	.05
**Current medication**		
Yes	-0.06 (-0.22 to 0.10)	.68
No	0.32 (0.18 to 0.46)	.01
**Comorbidity**		
**Anxiety**		
Yes	-0.15 (-0.35 to 0.05)	.52
No	0.09 (-0.03 to 0.20)	.48
**Depression**		
Yes	-0.03 (-0.21 to 0.16)	.90
No	0.06 (-0.06 to 0.19)	.60
**Somatic symptoms**		
Yes	0.10 (-0.06 to 0.27)	.54
No	0.07 (-0.07 to 0.20)	.61
**COVID-19**		
Before	-0.03 (-0.15 to 0.10)	.85
After	0.24 (0.07 to 0.41)	.22
**Overall**	**0.05 (-0.05 to 0.15)**	**.66**

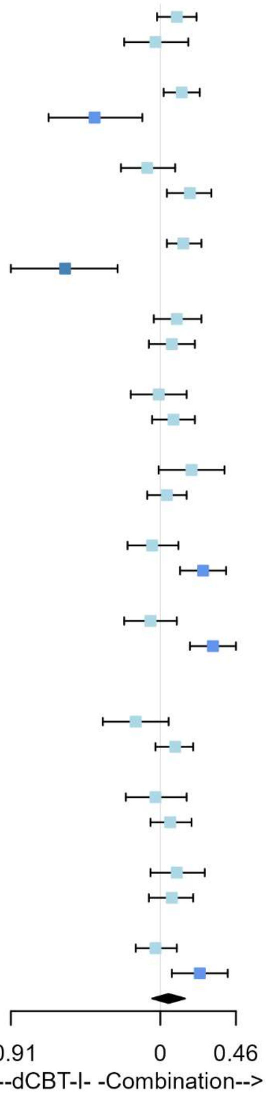

Each horizontal line on a forest plot represents the 95% confidence interval of comparison (Cohen's d effect size) of PSQI score at month 6 in a subgroup, and the result is plotted as a box in the middle of the horizontal line. The last row indicates the comparison of effectiveness on the whole population. The vertical line is a reference line, placed at the value where there is no difference between two interventions. The position of the box relative to the vertical line reflects the favorite (more effective) intervention mode. The box to the left of the vertical line indicates that the left treatment mode is superior to the right treatment mode shown at the bottom of the plot. Small, moderate, and large effect sizes are displayed in different colors, with darker colors indicating larger effect sizes. Education level and insomnia duration were coarsely categorized to avoid over-classification resulting in a smaller sample to influence confounders' control with IPTW. Patients with primary, middle and senior education were classified as low educated, while patients with higher education were classified as high educated.

Insomnia duration: Length of interval from the first occurrence of insomnia to baseline. First onset: Whether this insomnia was the first attack. Medication history: Whether the patient had a history of taking any form of hypnotic medications prior to baseline. Current medication: Whether the patient was still taking any form of hypnotic medications currently at baseline.

© 2023 Lu M et al. *JAMA Network Open.*

eReferences.

1. Qaseem A, Kansagara D, Forciea M, Cooke M, Denberg T, Amer Coll Phys. Management of chronic insomnia disorder in adults: A clinical practice guideline from the American college of physicians. *Ann Intern Med*. 2016;165(2):I26-I26. doi:10.7326/P16-9016
2. Edinger JD, Arnedt JT, Bertisch SM, Carney CE, Martin JL. Behavioral and psychological treatments for chronic insomnia disorder in adults: An American Academy of Sleep Medicine clinical practice guideline. *J Clin Sleep Med*. 2020;17(2).
3. Smyth C. The Pittsburgh sleep quality index (PSQI). *J Gerontol Nurs*. 1999;25(12):10-10.
4. Austin P. An introduction to propensity score methods for reducing the effects of confounding in observational studies. *Multivar Behav Res*. 2011;46(3):399-424. doi:10.1080/00273171.2011.568786
5. Nishida Y, Takahashi Y, Nakayama T, Soma M, Kitamura N, Asai S. Effect of candesartan monotherapy on lipid metabolism in patients with hypertension: A retrospective longitudinal survey using data from electronic medical records. *Cardiovasc Diabetol*. 2010;9. doi:10.1186/1475-2840-9-38
6. Feld E, Harton J, Meropol N, et al. Effectiveness of first-line immune checkpoint blockade versus carboplatin-based chemotherapy for metastatic urothelial cancer. *Eur Urol*. 2019;76(4):524-532. doi:10.1016/j.eururo.2019.07.032
7. Hatakeyama S, Tanaka T, Ikehata Y, et al. Axitinib versus sunitinib as first-line therapies for metastatic renal cell carcinoma: A multicenter retrospective analysis. *J Clin Oncol*. 2019;37(7). doi:10.1200/JCO.2019.37.7_suppl.555
8. van Duin D, Lok JJ, Earley M, et al. Colistin versus Ceftazidime-Avibactam in the treatment of infections due to carbapenem-resistant enterobacteriaceae. *Clin Infect Dis*. 2018;66(2):163-171. doi:10.1093/cid/cix783
9. El-Galaly A, Nielsen P, Kappel A, Jensen S. Reduced survival of total knee arthroplasty after previous unicompartmental knee arthroplasty compared with previous high tibial osteotomy: A propensity-score weighted mid-term cohort study based on 2,133 observations from the Danish Knee Arthroplasty Registry. *Acta Orthop*. 2020;91(2):177-183. doi:10.1080/17453674.2019.1709711
10. Zhang Z, Kim H, Lonjon G, Zhu Y, AME Big-Data Clinical Trial Collab. Balance diagnostics after propensity score matching. *Ann Transl Med*. 2019;7(1). doi:10.21037/atm.2018.12.10
11. Troxel W, Conrad T, Germain A, Buysse D. Predictors of treatment response to brief behavioral treatment of insomnia (BBTI) in older adults. *J Clin Sleep Med*. 2013;9(12):1281-1289. doi:10.5664/jcsm.3270
12. Wu Y, Yang L, Zhong Z, et al. Auricular acupressure for hemodialysis patients with insomnia: A multicenter double-blind randomized sham-controlled trial. *J Integr Complement Med*. doi:10.1089/jicm.2021.0332
13. Kang S, Kang J, Cho S, et al. Cognitive behavioral therapy using a mobile application synchronizable with wearable devices for insomnia treatment: A pilot study. *J Clin Sleep Med*. 2017;13(4):633-640. doi:10.5664/jcsm.6564
14. Kroenke K, Spitzer R, Williams J. The PHQ-9 - Validity of a brief depression severity measure. *J Gen Intern Med*. 2001;16(9):606-613. doi:10.1046/j.1525-1497.2001.016009606.x
15. Lakens D. Calculating and reporting effect sizes to facilitate cumulative science: a practical primer for t-tests and ANOVAs. *Front Psychol*. 2013;4. doi:10.3389/fpsyg.2013.00863
16. Cohen J. Statistical power analysis for the behavioral sciences. *J Am Stat Assoc*. 1988;2nd(334).

© 2023 Lu M et al. *JAMA Network Open.*
