# Supplementary material for: Comparative Effectiveness of Digital Cognitive Behavioral Therapy vs Medication Therapy Among Patients With Insomnia
Source: JAMA Netw Open. 2023 Apr 11;6(4):e237597. doi: 10.1001/jamanetworkopen.2023.7597 (PMC10091171; doi:10.1001/jamanetworkopen.2023.7597)
Supplement: Supplement 2. — Data Sharing Statement [file jamanetwopen-e237597-s002.pdf]

Data Sharing Statement

Lu. Comparative Effectiveness of Digital Cognitive Behavioral Therapy vs Medication Therapy Among Patients With Insomnia. *JAMA Netw Open*. Published April 11, 2023.
doi:10.1001/jamanetworkopen.2023.7597

Data
Data available: No
